# Pausing guides RNA folding to populate transiently stable RNA structures for riboswitch-based transcription regulation

**DOI:** 10.7554/eLife.21297

**Published:** 2017-05-25

**Authors:** Hannah Steinert, Florian Sochor, Anna Wacker, Janina Buck, Christina Helmling, Fabian Hiller, Sara Keyhani, Jonas Noeske, Steffen Grimm, Martin M Rudolph, Heiko Keller, Rachel Anne Mooney, Robert Landick, Beatrix Suess, Boris Fürtig, Jens Wöhnert, Harald Schwalbe

**Affiliations:** 1 Center for Biomolecular Magnetic Resonance, Institute of Organic Chemistry and Chemical Biology, Johann Wolfgang Goethe-University Frankfurt am Main, Frankfurt am Main, Germany; 2 Department of Biology, Technical University Darmstadt, Darmstadt, Germany; 3 Center for Biomolecular Magnetic Resonance, Institute of Molecular Biosciences, Johann Wolfgang Goethe-University Frankfurt am Main, Frankfurt am Main, Germany; 4 Department of Biochemistry, University of Wisconsin–Madison, Madison, United States; University of Toronto, Canada

**Keywords:** RNA, riboswitches, meta-stable structures, folding, transcription, kinetics, *B. subtilis*, *E. coli*

## Abstract

In bacteria, the regulation of gene expression by cis-acting transcriptional riboswitches located in the 5'-untranslated regions of messenger RNA requires the temporal synchronization of RNA synthesis and ligand binding-dependent conformational refolding. Ligand binding to the aptamer domain of the riboswitch induces premature termination of the mRNA synthesis of ligand-associated genes due to the coupled formation of 3'-structural elements acting as terminators. To date, there has been no high resolution structural description of the concerted process of synthesis and ligand-induced restructuring of the regulatory RNA element. Here, we show that for the guanine-sensing *xpt-pbuX* riboswitch from *Bacillus subtilis*, the conformation of the full-length transcripts is static: it exclusively populates the functional off-state but cannot switch to the on-state, regardless of the presence or absence of ligand. We show that only the combined matching of transcription rates and ligand binding enables transcription intermediates to undergo ligand-dependent conformational refolding.

**DOI:** http://dx.doi.org/10.7554/eLife.21297.001

## Introduction

Riboswitches are cis-regulatory RNA elements controlling cellular processes, in particular transcription and translation (Mironov et al., 2002; Nahvi et al., 2002; Mandal et al., 2003). They exert their function via a conformational switch between mutually exclusive base-paired structures. This conformational switch is induced by binding to a low-molecular weight ligand (Batey et al., 2004; Noeske et al., 2005; Serganov et al., 2004).

Riboswitches regulating transcription control gene expression by connecting ligand-dependent and ligand-independent RNA folding processes with the concomitant synthesis of the RNA by the RNA polymerase.

It has been increasingly recognized that the folding of RNA is intimately linked to transcription kinetics, which are directly dependent on transcriptional pausing. The synthesis of the RNA is not performed at a constant speed (Maizels, 1973; Darlix and Horaist, 1975). Instead, slow conformational changes, from a more competent to a less competent transcription state, lead to a variation of the transcription speed of the elongation complex (EC) between 2–30 nt/s (Davenport et al., 2000; Neuman et al., 2003). The less competent transcription state can be adopted at any point during transcription and can slow down the EC by inhibiting nucleotide addition (Adelman et al., 2002; Kireeva and Kashlev, 2009; Strobel and Roberts, 2015; Weixlbaumer et al., 2013). Thus, transcription rates vary during transcription. This elemental pausing occurs frequently, but typically lasts only for 1–6 s on average, making the overall transcription movement the sum of more and less competent transcription states averaged over the number of RNA polymerases (RNAP) molecules observed. In contrast to short elemental pausing, long pausing events occur at defined template sequences (pause sites) or upon interaction with protein factors (Roberts et al., 1998). At pause-sites, the RNAP speed decreases by a factor of 100 compared to its maximal transcription speed (max. 75 nt/s, measured on a specialized DNA template) (Vogel and Jensen, 1994). Pause sites can slow RNAP through backtracking of the enzyme relative to the nucleic acids or by the formation of a pause RNA hairpin after the EC has entered an initial elemental pause state which makes elemental pausing an obligatory intermediate from which longer pausing can occur (Reeder and Hawley, 1996; Kireeva et al., 2000; Artsimovitch and Landick, 2000; Landick, 2006; Nudler, 2012; Hein et al., 2014).

Pausing can also facilitate the folding of certain RNA structures and allow a stabilizing interaction between the nascent RNA and the transcription machinery (Pan et al., 1999). Pausing can be important for effective riboswitch-mediated regulation where co-transcriptional folding is influenced by the transcription speed (Wickiser et al., 2005; Perdrizet et al., 2012). The modulation of the available conformational space by altering the transcriptional speed was demonstrated for the *btuB* riboswitch where the use of a mutant RNA polymerase with deficient pause behavior caused the riboswitch to adopt a non-native structure and to be deficient in ligand binding (Perdrizet et al., 2012).

Here, we investigate the *xpt-pbuX* riboswitch from *Bacillus* and identify and characterize conformational sub-states and the kinetics of their inter-state conversion. Our work provides insight into how RNA polymerase residence time and the intramolecular refolding kinetics must be fine-tuned to allow riboswitch-based regulation of transcription. In this contribution, we apply high resolution structural and kinetic techniques to decipher the full co-transcriptional folding pathway, enabling monitoring the behaviour of the decisive structural units during synthesis for the first time.

### Results

**Figure 1.**
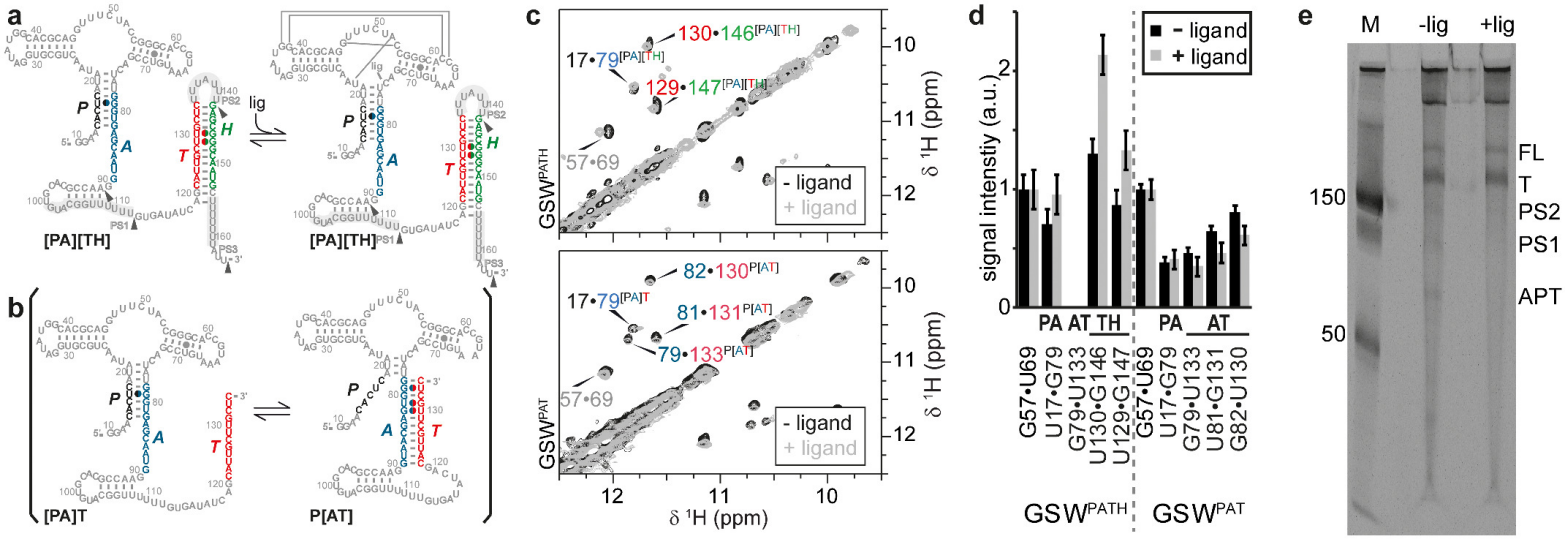
Conformational states of the guanine-sensing riboswitch depending on transcript length. (**a**) Secondary structure of the full-length guanine-sensing riboswitch (GSW^PATH^). In absence (*left*) and in presence of ligand (*right*) the aptamer closing helix (PA) and the terminator helix (TH) are formed, the anti-terminator helix (AT) is not present in either state. The only structural difference between the apo- and holo-states is the formation of a stable ligand binding pocket in the holo-state The strands involved in the switching mechanism are colour-coded: aptamer strand (P, *black*), aptamer stabilizing strand (A, *blue*), switching strand (T, *red*), and terminator strand (H, *green*). Putative pause sites (PS1-PS3) are indicated, the sequence highlighted in grey is occupied by the polymerase (Monforte et al., 1990); additionally, stable structured fragments are marked by arrows; (**b**) Secondary structure of a truncated stably structured guanine-sensing riboswitch (GSW^PAT^). In the absence of ligand two conformational states are populated in a 1:1 ratio each representing a functional (on/off) state of the riboswitch, (**c**) G·U region of NOESY spectra of the full-length GSW^PATH^ (*upper panel*) and the truncated riboswitch GSW^PAT^ (*bottom*). G·U cross peaks are reporters for formation of PA (black/*blue*), P3 (*grey*), AT (blue/*red*) and TH (red/*green*), respectively; (**d**) signal intensities of GSW^fl^ and GSW^10-134^ NOESY cross peaks in absence and presence of ligand. Errors were estimated from the noise of the respective spectra. The full-length riboswitch GSW^PATH^ adopts the terminator conformation irrespective of the ligand. The truncated GSW^PAT^ shows a heterogeneous fold in the absence and in the presence of the ligand, (**e**) 10% PAGE of the overnight transcription of the full length riboswitch in the absence (-lig) and presence (+lig) of ligand. The transcribed RNA fragments correspond to the full length (FL: 228 nt), the Terminator (T: 172 nt), the second pause site (PS2: 152 nt), the first pause site (PS1: 124 nt) and the aptamer (APT: 96 nt). **DOI:** http://dx.doi.org/10.7554/eLife.21297.002

The guanine-sensing riboswitch from *Bacillus subtilis* (GSW) negatively regulates the transcription of the *xpt*-gene by the premature termination of RNA synthesis (Mandal et al., 2003). In this riboswitch, the regulatory output signals of maintaining or repressing transcription require two conformational states with different base-pairing interactions of four complementary sequences P, A, T and H (Figure 1a). In the off-state, the 5’-aptamer-strand (P) pairs with the aptamer-stabilising strand (A) and the switching strand (T) forms the terminator helix together with the terminator strand (H). By contrast, in the putative on-state (Figure 1b), A and T pair and form the antiterminator conformation, and the P and H strands are unpaired.

**Figure 1—figure supplement 1.**
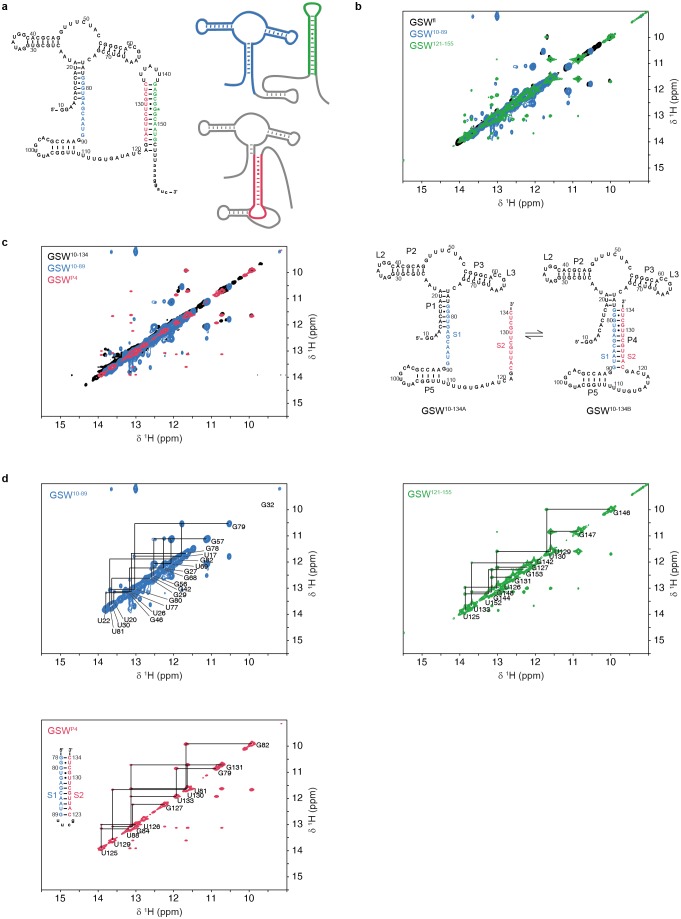
Assignment of GSW constructs. All ^1^H,^1^H-NOESY spectra were recorded at 283 K in 2 mM magnesium chloride, 50 mM potassium chloride, 25 mM potassium phosphate (pH 6.2). (**a**) Module design for chemical shift assignment of the full-length GSW with in the divide and conquer approach. The full-length GSW and the modules were measured by NMR spectroscopy seperately. *Left*: GSW^fl^ (GSW^PATH^*, grey*) in the terminator conformation, the aptamer domain GSW^10-89^ (GSW^PA^, *blue*) and the terminator hairpin GSW^121-155^ (TH, green) are indicated. *Right*: GSW^fl^ (GSW^PATH^, *grey*) in the antiterminator conformation and the P4 module (AT, *red*). (**b**) Overlay of the full-length GSW^fl^ (GSW^PATH^, *black*), the aptamer domain GSW^10-89^ (GSW^PA^, *blue*) and the terminator hairpin GSW^121-155^ (TH, *green*) shows that GSW^fl^ (GSW^PATH^) adopts the terminator conformation with formed aptamer and terminator hairpin. Assignment of the fragments can be transferred to GSW^fl^ (GSW^PATH^). (**c**) *Left*: Overlay of the truncated GSW^10-134^ (GSW^PAT^, *black*), the aptamer domain GSW^10-89^ (GSW^PA^, *blue*) and the P4 module (AT, *red*) reveals conformational heterogeneity of GSW^10-134^ (GSW^PAT^)with either helix P1(PA) (GSW^10-134A^, GSW^[PA]T^) or helix P4(AT) (GSW^10-134B^, GSW^P[AT]^) formed. *Right*: Conformations GSW^10-134A^ (=GSW^[PA]T^) and GSW^10-134B^ (=GSW^P[AT]^) of the truncated GSW^10-134^ (=GSW^PAT^). (**d**) Spectra of the aptamer domain GSW^10-89^ (GSW^PA^, *blue*), the terminator hairpin GSW^121-155^ (TH, *green*) and the P4 module (AT, *red*). Assignments are annotated in the spectra; the sequence of the P4 (AT) module is given. **DOI:** http://dx.doi.org/10.7554/eLife.21297.003

**Figure 1—figure supplement 2.**
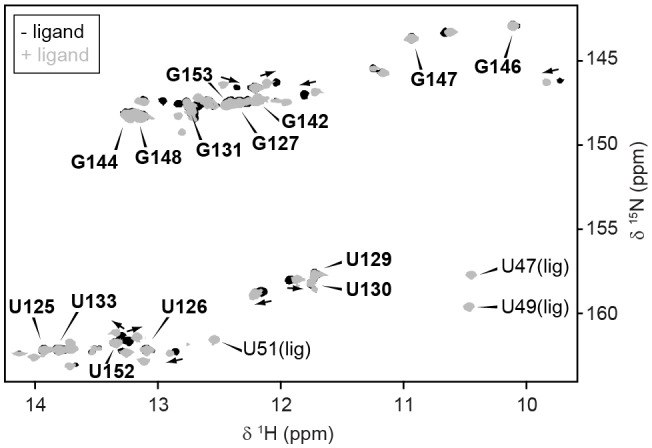
Conformation of full-length GSW. GSW^fl^ without (*black*) and with ligand (*grey*) adopts the terminator conformation. Experimental conditions are 2 mM magnesium chloride, 50 mM potassium chloride, 25 mM potassium phosphate (pH 6.2). The resonance assignment of the terminator hairpin P6 is depicted in *bold letters*. In presence of ligand, reporter signals (U47, U49 and U51) from the binding pocket are detected. However, ligand binding induces slight chemical shift changes (*arrows*) but no conformational changes are observed in particular not for TH. **DOI:** http://dx.doi.org/10.7554/eLife.21297.004

The free energy landscape of RNA folding features high barriers for the transitions between conformations with alternative base-pairing. These transitions are frequently found to be slower than the timescale of the relevant biological process (Solomatin et al., 2010). In addition, RNA chains can be trapped in a stable conformation (Treiber et al., 1998). The terminator conformation of GSW represents such a ‘super’ stabilised structure. Regardless of the presence or absence of the cognate ligand, the RNA adopts a single conformation in which the terminator helix (TH) is formed as inferred from NMR signals (for assignment, see Figure 1 and Figure 1—figure supplement 1) arising from the stable GU base-pairs (U129-G147 and U130-G146) (Figure 1c,d). Addition of the ligand to the GSW induces only changes within the binding core of the aptamer domain (Noeske et al., 2007) but the aptamer closing helix (PA) is stably present both in the apo and holo form of the GSW and formation of the antiterminator helix (AT) cannot be detected (Figure 1c,d; Figure 1—figure supplement 2).

Since the terminator conformation in the full-length construct is the only detectable state regardless of the presence or absence of ligand, it must be stabilised by at least △△G > 8 kJ mol^−1^ compared to the antiterminator conformation at a NMR signal-to-noise greater than 10:1.

These observations give rise to two fundamentally important questions: If the full-length riboswitch is trapped in a single stable structure and not able to refold, which transcription intermediate is capable of undergoing the regulatory active conformational change (Wickiser et al., 2005; Gilbert et al., 2006; Lemay et al., 2006)? If the thermodynamic stable conformation resembles an off-state, how can the organism ever adopt the on-state to allow gene expression?

We therefore tested for accumulation of distinct transcription intermediates, occurring as shorter RNA products are produced during the synthesis of full-length riboswitches (Artsimovitch and Landick, 2000). In multiple round transcription reactions, we detected several distinct shorter RNA products (Figure 1e). Three of the detected fragments correspond to the aptamer domain (nucleotides 10–89), to the prematurely terminated form (nucleotides 10–164), and to the full-length of the mRNA-5’-UTR. Mapping of these RNA fragments to the riboswitch structure reveals that they correspond to transcription intermediates that include the strands A, T, and H at their 3’-ends, respectively. Furthermore, two additional fragments of 100 and 140 nucleotides are detected. These latter RNAs exhibit 3’-terminal stretches of U-residues and therefore represent canonical transcriptional pause sites (Landick, 2006; Larson et al., 2014).

**Figure 2.**
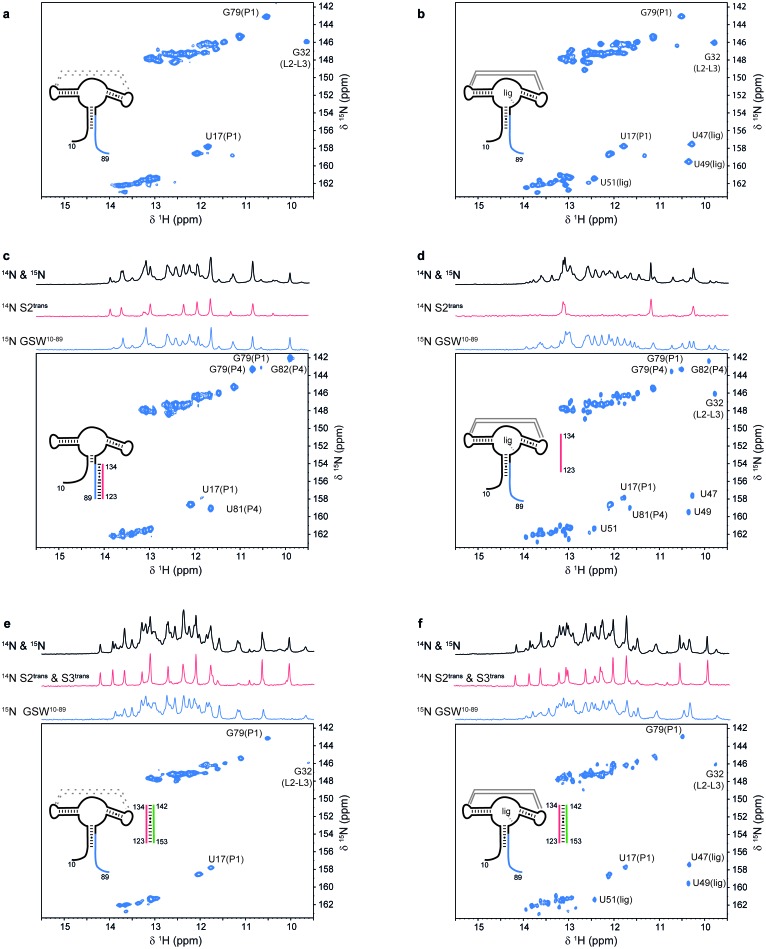
Models for intermediates during transcription. All NMR spectra were recorded at 283 K in 2 mM MgCl_2_, 50 mM KCl, 25 mM potassium phosphate (pH 6.2). 1 equivalent of each RNA (GSW^10-89^ (=GSW^PA^), S2^trans^ (=T^trans^) and S3^trans^(=H^trans^), respectively) was used, 4 equivalents of ligand were added. The following selective labelling scheme was explored: ^15^N-G,U GSW^10-89^, ^14^N-G,U S2^trans^ and ^14^N-G,U S3^trans^. Signals originating from GSW^10-89^ and S2^trans^ or S3^trans^ were separated using in x-filter 1D experiments (Weixlbaumer et al., 2013) (*top*). The ^1^H,^15^N-HSQC spectra (*bottom*) report on the interactions in the aptamer domain. (**a**) In the elongated aptamer domain GSW^10-89^, the helix PA was formed (U17–G79) and the loop-loop interaction reporter G32 was detected. (**b**) GSW^10-89^ and ligand (1:4) ligand binding was monitored by appearance of signals U47, U49 and U51. (**c**) GSW^10-89^ and S2^trans^ (1:1): Sequence S2^trans^ caused the PA reporter signals U17(P1) and G79(P1) to decrease, AT formation was followed by appearance of signals of G79(P4), U81(P4) and G82(P4). (**d**) GSW^10-89^, S2^trans^ and ligand (1:1:4): Addition of ligand to GSW^10-89^-S2^trans^ resulted in dissociation of the complex (decreasing signals for G79(P4), U81(P4) and G82(P4) signals) and reformation of the PA helix (U17(P1) and G79(P1) signals). Ligand binding reporters U47, U49 and U51 were detected. However, in presence of 4 equivalents of ligand, AT helix reporter signals were significant. (**e**) GSW^10-89^, S2^trans^ and S3^trans^ (1:1:1) Addition of S3^trans^ to GSW^10-89^-S2^trans^ resulted in complete dissociation of the complex (G79(P4), U81(P4) and G82(P4) signals) and reformation of the PA helix (U17(P1) and G79(P1) signals). In contrast to ligand addition, 1 equivalent of S3^trans^ was sufficient to disrupt the antiterminator mimic. (**f**) GSW^10-89^, S2^trans^, S3^trans^ and ligand (1:1:1:4): Ligand binding to GSW^10-89^ in presence of the terminator helix P6 (=TH) equals ligand binding to GSW^10-89^ (GSW^PA^) alone (**b**). **DOI:** http://dx.doi.org/10.7554/eLife.21297.005

Based on the detection of these fragments, we truncated GSW after strands A or T to generate transcription intermediates containing either only the aptamer domain (GSW^PA^) or the aptamer domain and the subsequent sequence region of the antiterminator (GSW^PAT^). Both GSW fragments bind the ligand with similar affinities as the full-length riboswitch suggesting formation of the unperturbed aptamer domain (Figure 2 and Figure 2—figure supplement 1).

**Figure 2—figure supplement 1.**
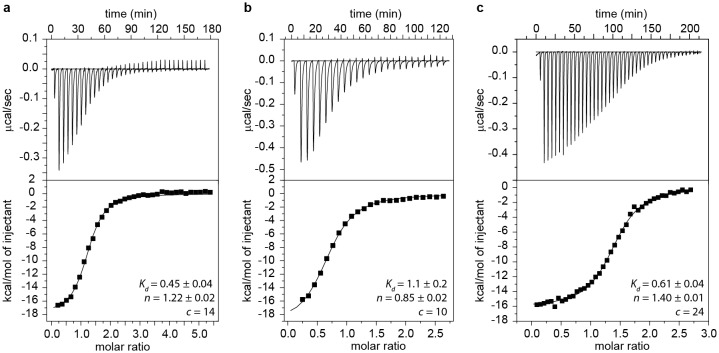
ITC measurements and *K*_d_ values of GSW^PA^ (**a**), GSW^PAT^ (**b**) and GSW^PATH^ (**c**). ITC measurements were performed with a Microcal VP ITC (Northampton, MA USA) at 10°C. A 217 μM solution of ligand (hypoxanthine) was titrated to a 15 μM solution of RNA using 25–42 injections. Buffer conditions were 2 mM magnesium chloride, 50 mM potassium chloride, 25 mM potassium phosphate, pH 6.2. The data was analyzed with the Origin ITC software (OriginLab, Northampton, MA USA) assuming a single binding site. The Kd values are given in µM. **DOI:** http://dx.doi.org/10.7554/eLife.21297.006

For GSW^PAT^, we observed two equally intense sets of NMR signals from two long-lived conformations GSW^[PA]T^ and GSW^P[AT]^ (Figure 1b,c) that differ with regard to the formation of the two mutually exclusive helices PA or AT. In GSW^[PA]T^, helix PA is formed, and in GSW^P[AT]^ the antiterminator helix AT is formed. Upon addition of ligand, we detect ligand binding and subsequent shifting of the conformational equilibrium towards GSW^[PA]T^ (Figure 1c,d). Thus, in contrast to the full-length riboswitch, the antiterminator conformation representing the functional on-state is a thermodynamically stable state (GSW^P[AT]^) of this transcription intermediate (GSW^PAT^) in the absence of ligand.

In order to evaluate ligand binding and the refolding kinetics of transcriptional intermediates, we mimicked these processes by sequentially adding short oligonucleotides of sequences T (T^trans^) and H (H^trans^) to GSW^PA^. Using isotope-filter NMR experiments, selectively ^15^N-labelled GSW^PA^ (containing the P and A sequences), and ^14^N-labelled T^trans^ and H^trans^ we investigated the sequential refolding of the mRNA transcription intermediates with nucleotide resolution (Figure 2). The imino-signal stemming from the aptamer domain in the ligand-free (Figure 2a) and –bound state (Figure 2b) can be readily assigned and constitute the characteristic NMR signals to detect the relevant GSW conformations and their ligand-induced refolding. Without ligand, addition of equimolar amounts of T^trans^ to GSW^PA^ led to formation of the antiterminator conformation mimic containing helix AT and the strand P is unpaired (Figure 2c). This GSW^PA^-T^trans^ complex was disrupted by the addition of ligand (Figure 2d), restoring formation of helix PA. In contrast, the presence of ligand stabilised GSW^PA^ so that subsequent addition of T^trans^ did not disrupt helix PA in the aptamer domain within the GSW^PA^-ligand-complex and formation of the antiterminator helix was suppressed. From these findings, we can conclude that ligand binding to GSW^PA^ represents the structural decision point.

Regardless of the presence or the absence of ligand, addition of H^trans^ to the antiterminator conformation in the GSW^PA^-T^trans^ complex induced a conformational switch characterized by the formation of the terminator helix TH and the helix PA (Figure 2e and f).

**Figure 3.**
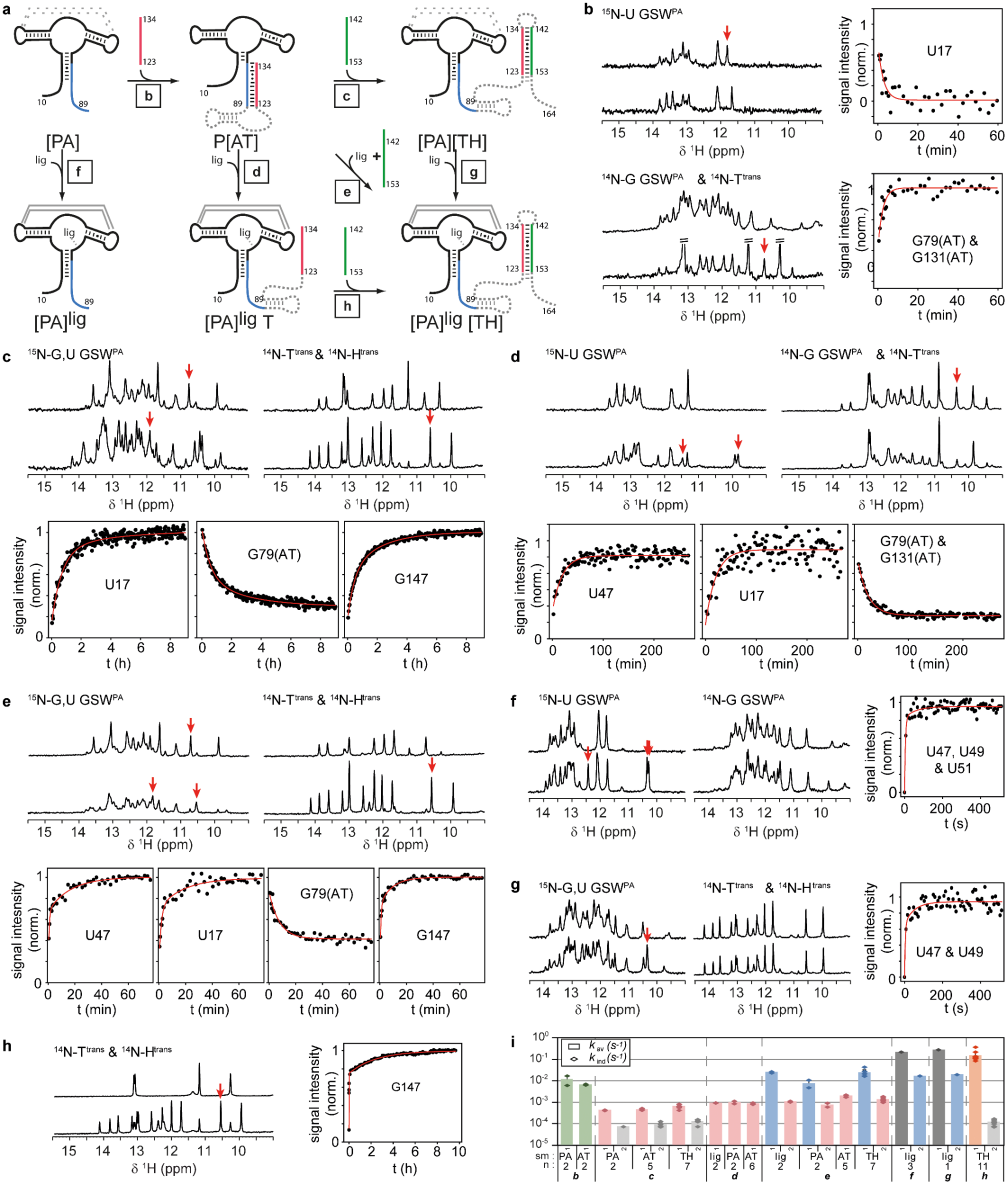
RNA refolding and ligand binding kinetics. (**a**) Schematic overview of the performed kinetic experiments (indicated with letters b-h) to characterise different transcription intermediates. The aptamer domain GSW^PA^ is depicted in *black*, switching sequences A (*blue*), T (*red*) and H (*green*) are colour-coded. Dashed sequences are neglected in the antisense oligonucleotide approach. (**b–h**) results of the kinetic experiments, lettering according to a). ^15^N- (*left*) and ^14^N-filtered (*right*) 1D spectra before (*top*) and after (*bottom*) the kinetic experiments are depicted. An exemplary time trace reporting on the formation of each structural motif involved in the rearrangement is given, respective signals are marked with red arrows. (**i**) Rates obtained from signal traces of resolved imino proton resonances. Letters refer to the kinetic experiments as shown in **a**). For all structure motifs (ligand binding, PA, AT and TH formation, respectively) several signals (number indicated below) were analysed, averaged rates are given in *bars*, rates of individual base pairs are indicated with *diamonds*. Colour-coding refers to AT association (*green*), AT dissociation (*red*), ligand binding (*dark grey*), aptamer formation (*blue*), terminator association (*orange*) and H^trans^-intrinsic unfolding (*light grey*) which is irrelevant for riboswitch function. Residues with a single rate were fitted mono-exponentially, a bi-exponential fit function was applied for residues with two distinguishable rates. For exact values and errors see Supplementary file 1. **DOI:** http://dx.doi.org/10.7554/eLife.21297.007

Having characterized these three functional relevant states, we conducted real-time NMR experiments (Buck et al., 2009) to determine the kinetics of RNA refolding to the antiterminator and to the terminator conformation and of ligand binding to the distinct conformations, i.e. the critical events for GSW function (Figure 3). Utilizing the above described labeling strategy, we were able to resolve the NMR signals from the ligand, the acceptor domain and the associating or dissociating single RNA strand. In total, 59 signals could be detected, and the reported kinetics have been measured in triplicate experiments and averaged over structural elements in the riboswitch.

**Figure 3—figure supplement 1.**
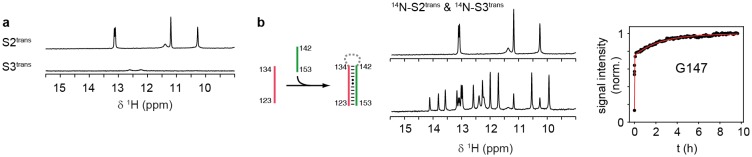
Folding of sequences T^trans^ and H^trans^. (**a**) Imino proton region of the ^1^H-NMR spectra of the oligonucleotide sequences T^trans^ (*top*) and H^trans^ (*bottom*). NMR spectra were recorded at 283 K in 2 mM magnesium chloride, 50 mM potassium chloride, 25 mM potassium phosphate (pH 6.2). The sequence T^trans^ shows intrinsic folding, intrinsic interactions of H^trans^ are minor. **DOI:** http://dx.doi.org/10.7554/eLife.21297.008

**Figure 3—figure supplement 2.**
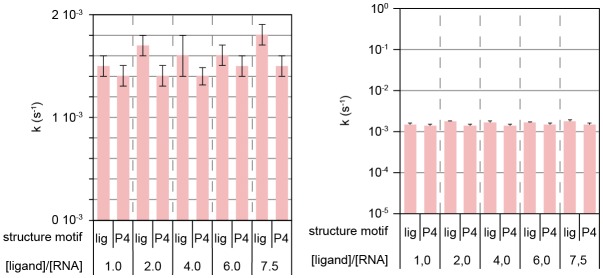
Ligand-independent dissociation of helix AT in kinetic experiment d. Rates obtained from signal traces of resolved imino proton resonances. **DOI:** http://dx.doi.org/10.7554/eLife.21297.009

Ligand binding needs to lock strand A in the aptamer’s PA helix and suppress formation of the antiterminator conformation. Indeed, ligand binding to GSW^PA^ either in the absence or in presence of the terminator helix (Figure 3f and g) was fast, with a *k*_1_ of (2.4 ± 0.3) 10^−1^ s^−1^, and induced a slower tertiary structure rearrangement (*k*_2_ = [1.8 ± 0.2) 10^−2^ s^−1^] within the aptamer, consistent with previous experiments (Vogel and Jensen, 1994; Reeder and Hawley, 1996). Subsequent formation of the terminator helix from single strands was also rapid (*k*_1_ = [1.4 ± 0.8] 10^−1^ s^−1^; Figure 3h).

In the absence of ligand, GSW^PA^ and T^trans^ anneal rapidly to form the antiterminator complex (*k* = [9 ± 5] 10^−3^ s^−1^; Figure 3b). Subsequent formation of the terminator helix TH was observed if H^trans^ was added to the GSW^PA^-T^trans^ complex (antiterminator conformation) (Figure 3c). Under these conditions, the dissociation of the antiterminator helix AT was rate-limiting and slow (*k*_1_ = [0.5 ± 0.2] 10^−3^ s^−1^; *k*_2_ = (1.1 ± 0.4) 10^−4^ s^−1^[T^trans^ intrinsic unfolding, Figure 3—figure supplement 1]). Importantly, the dissociation of helix P4 was accelerated by the presence of ligand, but in a concentration-independent manner above equimolar ratios (Figure 3—figure supplement 2; *k* = [0.89 ± 0.09] 10^−3^ s^−1^; Figure 2d) and was stimulated to a higher degree if ligand binding and annealing of H^trans^ occurred simultaneously (Figure 3e). Under the latter conditions, the formation of the terminator conformation was described by two rate constants, *k*_1_ = (2 ± 1) 10^−2^ s^−1^ reporting on the ligand-induced refolding of the aptamer domain and *k*_2_ = (1.2 ± 0.4) 10^−3^ s^−1^ characterising the rate-limiting dissociation of helix AT.

From these kinetic data (Supplementary file 1, Figure 3i), we conclude that the AT conformation represents a meta-stable state that slowly refolds into the thermodynamically stable terminator state (which represents the state of lowest free energy). The dissociation of PA is slow compared to the polymerase transcription speed. Therefore, during mRNA synthesis, transcriptional resting states are required to slow transcription to ensure sufficient time for the refolding of conformations [PA]T to P[AT] to enable riboswitch function. We are aware that in this applied experimental strategy some of the kinetics are measured in trans, although the riboswitch is a cis-acting element. However, at the applied concentrations of 0.1 mM, the most relevant conformational transition of ligand-dependent [PA] is slow and if the rate may possibly be overestimated by this method, which would not impact the fundamental conclusion as discussed below.

**Figure 4.**
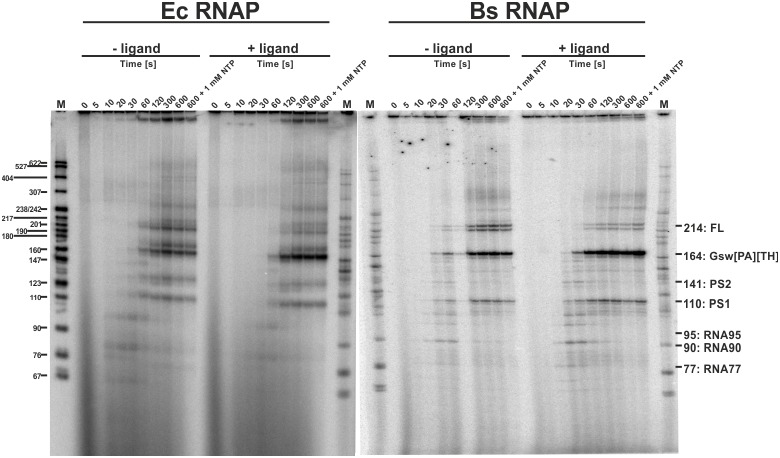
Time resolved Transcription: 8% PAGE of time resolved transcriptions using the *E.coli* (EC holo) and the *B. subtilis* (BS holo) RNAPs in the absence (-ligand) and presence (+ligand) of ligand. The time points of transcription stops are indicated in (s). The positions of ^32^P 5’-labeled markers (pBR322 MspI digested) are indicated on the left hand side. Prominent paused and terminated bands are indicated on the right hand side. seven major RNA-fragments could be identified: The run-off transcript or full-length RNA (FL), the premature termination fragment (Gsw^PATH^), the second pause-site (PS2), the first pause-site (PS1) and three pausing fragments (RNA95 and RNA77 for *E. coli* RNAP transcriptions and RNA90 and RNA77 for *B. subtilis* RNAP transcriptions). Over time, both RNAPs transcribe the DNA-template, generating RNA-fragments of increasing size. A pausing event is characterized by signal increase and by a fast increase of the signal followed by a slower decrease (e.g. RNA77). FL and Gsw^PATH^ show a strong accumulation over time and when ligand is added, the signal intensity of FL is decreased whereas the signal intensity of Gsw^PATH^ is increased. **DOI:** http://dx.doi.org/10.7554/eLife.21297.010

Transcription rates between 12 to 90 nucleotides per second have been reported in prokaryotes (Adelman et al., 2002; Vogel and Jensen, 1994). However, rates of synthesis vary during transcription. 5’-untranslated regions (5’-UTRs) for example often contain several U-rich sequences that have been shown to constitute transcriptional pause sites (Artsimovitch and Landick, 2000; Gusarov and Nudler, 1999). Other sequences can also induce pausing of RNA polymerase, in particular in *B. subtilis* (Larson et al., 2014). In any case, pausing has been reported to assist co-transcriptional folding e.g. of the FMN- and coenzyme B_12_-sensing riboswitches (Wickiser et al., 2005; Perdrizet et al., 2012). Within the sequence of GSW, a U_6_-stretch (U107-U112) is suspiciously located between the aptamer domain and the terminator helix (pause site 1, PS1) and between the A and T stretches of the antiterminator (U135-U141, pause site 2, PS2) (for analysis of the distance between aptamer and terminator sequences in purine riboswitches see Figure 4—figure supplement 1). We analysed the synthesis rate of the RNA polymerases form *E. coli* (EcRNAP) and *B. subtilis* (BsRNAP) in a time-resolved single-round transcription assay (Figure 4). Seven transcribed RNAs could be identified, suggesting that the formation of the shorter products after transcription of the aptamer domain and PS1 accumulate at a later time point during the overnight multi-round transcription. The seven RNA fragments were identified as FL (214 nt), GSW^PATH^ (164 nt), PS2 (141 nt), PS1 (between 107 and 112 nt, Figure 4—figure supplement 1), and three additional paused RNA fragments. Two of these additional pauses were seen with transcription with EcRNAP and were mapped as RNA95 and RNA77 (transcripts 95 nt and 77 nt in length, respectively). Transcription with BsRNAP through the same sequence showed the same pausing at RNA77, pausing at a different position, RNA90 (transcript 90 nt in length), but no pausing at RNA95. The FL and GSW^PATH^ fragments accumulated over time and reached their maximum at 300 s and more than 600 s, respectively, for both the Ec and Bs RNAPs. The GSW^PATH^ concentrations did not reach their maximal values within the observed 600 s. For the determination of transcription rates, the maximum values were included in the fitting procedure. Upon ligand addition, the intensity of GSW^PATH^ increases whereas the intensity of FL decreased. The intensities of the pausing transcription intermediates appear unaffected by ligand addition. The data indicate that the riboswitch maintains its ligand-response function during the in vitro transcription assays, as the termination efficiency is increased by 24.8% for EcRNAP and by 30.3% for BsRNAP. The intensities of the PS2 and PS1 RNA also show an exponential growth, reaching maximum value after 120 s, indicating that under low NTP-concentrations, the poly-U sequences act more as a polymerase blocking point than a pause-site. This could be observed for both RNAPs. The intensity of PS2 is near the level of noise for BsRNAP, making quantitation of the amount of transcript difficult for this fragment. However, the pausing values could be determined, and these are of higher importance for the simulations. Furthermore, the intensity of PS1 is stronger for BsRNAP transcription, indicating that PS1 has a higher influence on the elongation complex for the BsRNAP. In addition, the determined pausing-parameters (Supplementary file 1) show that both RNAPs pause longer at PS1 than at PS2. This effect is increased for the EcRNAP when ligand is added whereas pausing of the BsRNAP shows no ligand dependency. Relative to the pausing observed at PS1 and PS2, pausing at RNA77, RNA90, and RNA95 was of shorter duration for both RNAPs. For EcRNAP-mediated transcription in the absence of ligand, pausing at RNA77 could not be clearly determined as the relative intensities show a linear than an exponential decay. However, as four pausing events are observed for each RNAP, at least in the presence of ligand, even these brief pauses can have a high impact on the overall transcription kinetics. The apparent transcription rates were determined to range from 0.4 ± 0.1 nt s^−1^ and 2.0 ± 0.5 nt s^−1^ (FL and GSW^PATH^) to 1.3 ± 0.6 nt s^−1^ and 47.0 ± 53.3 nt s^−1^ for the synthesis of the shorter transcription intermediates at 37°C (Figure 4). Upon ligand addition, the apparent transcription rate decreased by at least a factor of two for PS2 and PS1 but was nearly identical for RNA95/RNA90 and RNA77. However, the multiple pausing events during transcription drastically decrease the apparent transcription rates of the longer RNAs FL and GSW^PATH^.

**Figure 4—figure supplement 1.**
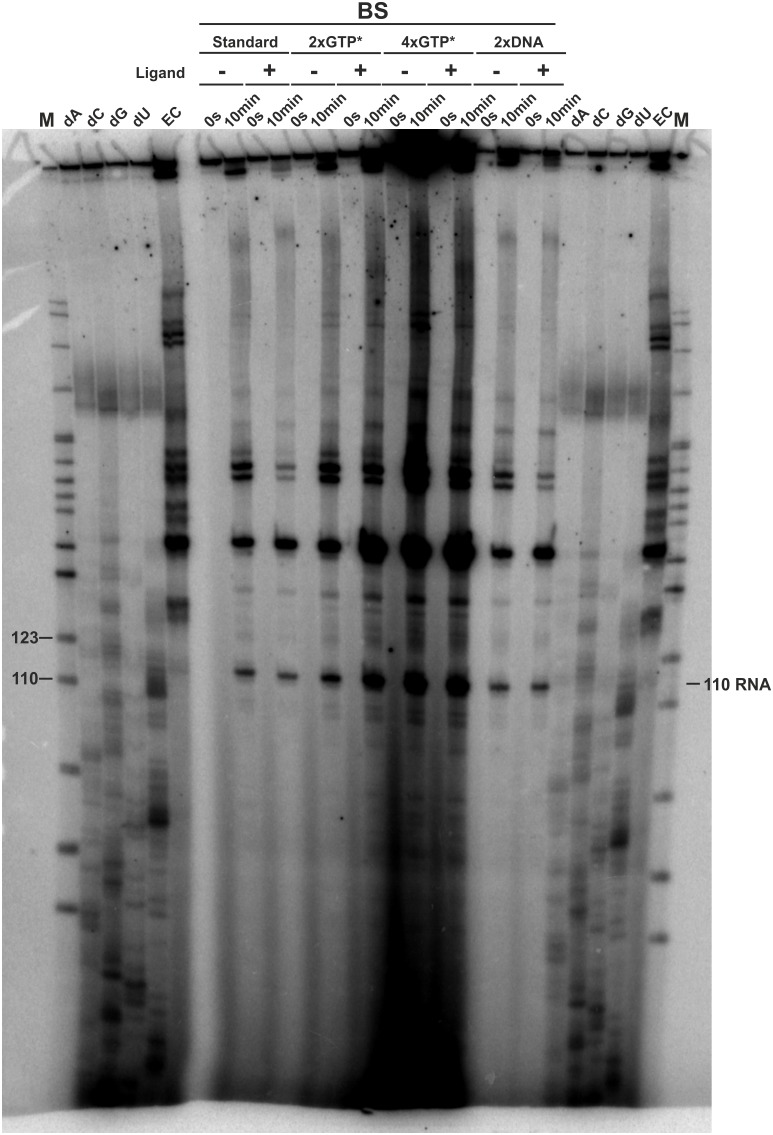
3’-mapping and GTP* increase. Transcription was performed using the *E. coli* RNAP in the absence (EC) and presence of 3’-deoxy ATP (dA), CTP (dC), GTP (dG) and UTP (dU), respectively and compared to transcriptions using the *B. subtilis* RNAP with different amounts of radioactively labeled GTP (GTP*) or DNA template in the absence and presence of ligand. The gel shows several transcription abortion products which end with a 3’-deoxy U and which migrate slightly faster than the 110 RNA fragment. When compared to the sequence, this poly-U stretch corresponds to the bases T107 to T112. However, it can’t be clearly stated on which nucleotide the 110 RNA ends. It was therefore decided to call this fragment 110. **DOI:** http://dx.doi.org/10.7554/eLife.21297.011

**Figure 4—figure supplement 2.**
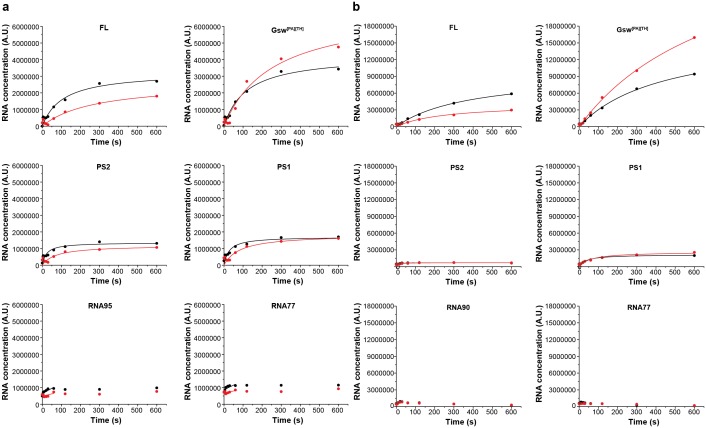
Intensity plots of the normalized pause signals. (**a**) The signal intensities of the *E. coli* RNAP transcripts PS2, PS2, RNA95 and RNA77 were analyzed as shown by Landick et al. (1996) in the absence (black) and presence (red) of ligand and plotted over time. For normalization, the intensity of an RNA signal (RNA) was divided by the sum of all RNAs of the same length and longer (RNAp). (**b**) Pausing plots of the *B. subtilis* RNAP transcripts. The steeper the pausing-plot, the shorter the dwell-time (τ) of the pause site. Pause-sites with high τ have a higher impact on transcription kinetics. Addition of ligand seems to have a higher impact on the pausing of the *E. coli* RNAP. However, the differences of the plots are within the errors. **DOI:** http://dx.doi.org/10.7554/eLife.21297.012

**Figure 4—figure supplement 3.**
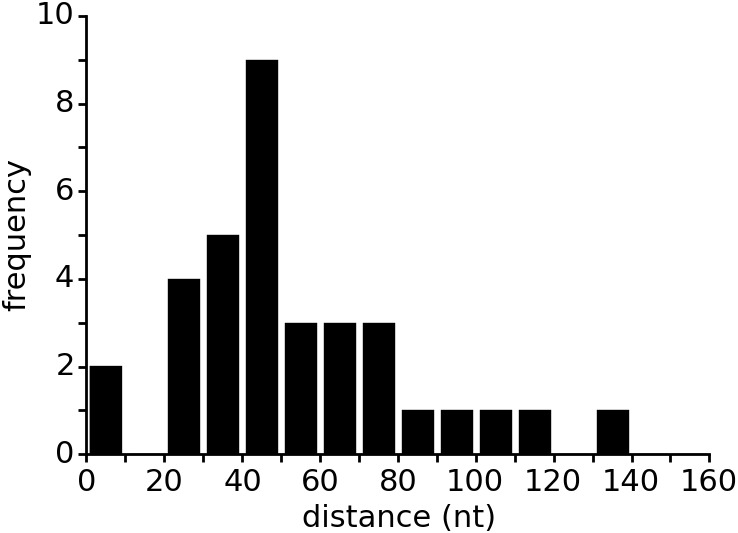
Distance between aptamer and terminator hairpin in purine riboswitches. The list of 133 riboswitches was taken from the seed dataset of the Rfam database entry for the Purine riboswitch family (RF00167). Riboswitch sequences were retrieved from the EMBL European Nucleotide Archive (ENA). The Rfam database contains only the aptamer regions of the purine riboswitches but lacks the expression platform. Therefore, the database does not annotate the terminator location. The web service ARNold, which uses Erpin (Gautheret and Lambert, 2001) and RNAmotif (Macke et al., 2001) to predict terminators, was run on all riboswitch sequences to determine the terminator position. Sequences upstream of the coding sequence (CDS) as identified by annotations in ENA were used as input for ARNold. ARNold identified terminators between the aptamer and the CDS in 35 of the 133 sequences. The distances between aptamer and terminator were calculated based on the secondary structure annotation from Rfam and the output from ARNold. Predicted terminators inside the aptamer sequence were excluded from the data. **DOI:** http://dx.doi.org/10.7554/eLife.21297.013

**Figure 5.**
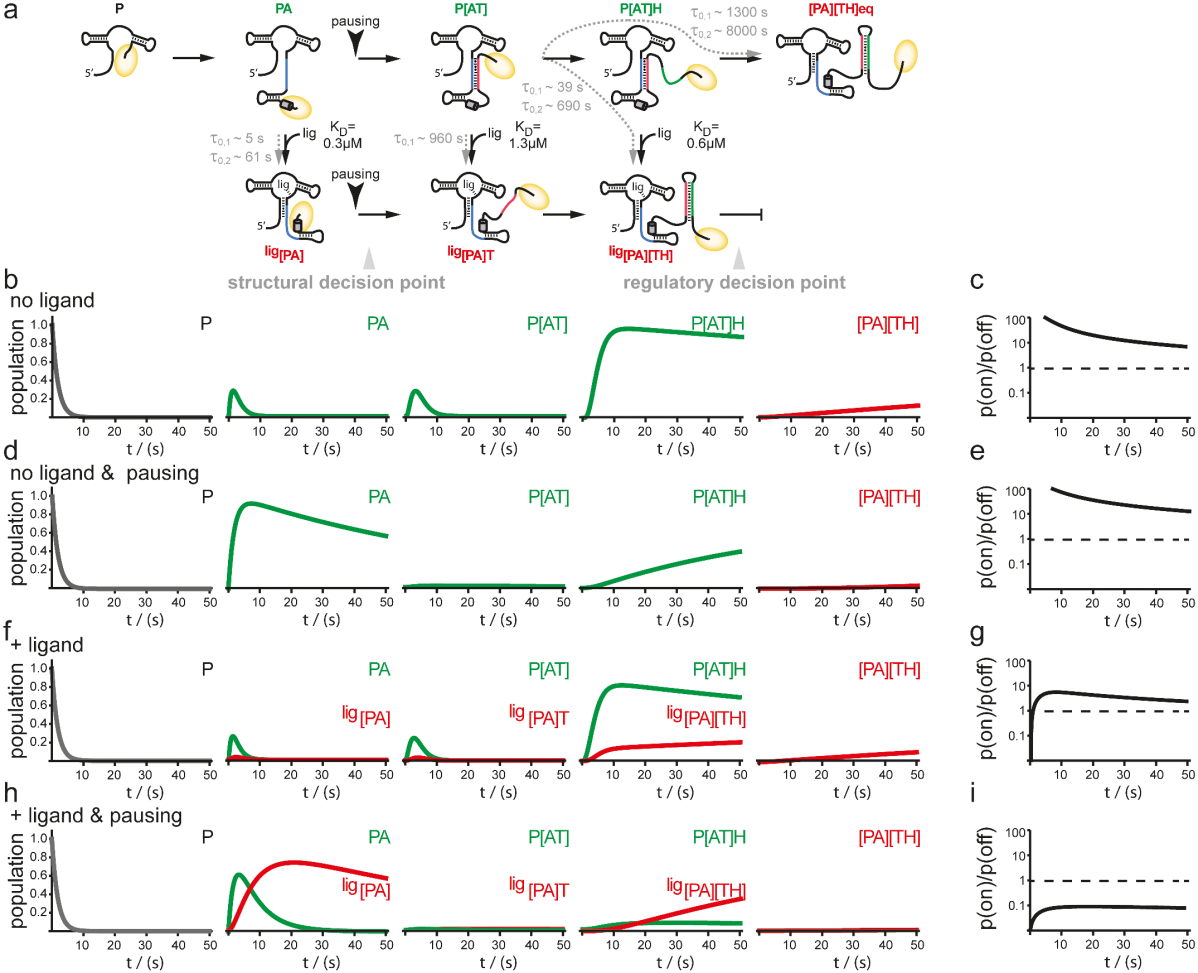
Simulation of co-transcriptional folding pathways. (**a**) Conformational states of GSW in context of the transcription progress are shown from left to right.* K*_d_ values of the transcript intermediates are indicated. Ligand binding can occur as soon as the aptamer domain is transcribed and consequently locks A (*blue*) in the PA helix resulting in the population of a single conformation during the transcription process and subsequently in transcription termination. The aptamer domain is synthesized first. As transcription continues, the free mRNA adopts the metastable antiterminator conformation stabilized by AT interaction (*blue* and *red*, respectively) which refolds to the terminator conformation after the riboswitch is completed. Pause site PS1 (*grey cylinder*) increases the available time window for ligand binding. Co-transcriptional refolding processes are indicated with their respective life times by the *black arrows*. Population of the conformational states under different conditions (the time plotted on the x-axis is the time of transcription with t = 0 at nucleotide position 75) (**b**) without ligand and without pausing, (**d**) without ligand and with pausing at PS1 and (**f**) with ligand and without pausing and (**g**) in presence of ligand and with pausing at PS1. Ratio of the probabilities to populate the on- versus the off-state as derived by kinetic simulation of the switching mechanism in absence (**c, e**) and in presence (**g, i**) of ligand. If the polymerase does not pause between the synthesis of the stretches A and T, GSW adopts the on-state irrespective of ligand (**c, g**) whereas pausing at PS1 enables GSW to respond to its ligand and dominantly adopts the off-conformation in the presence of ligand (**i**). **DOI:** http://dx.doi.org/10.7554/eLife.21297.014

In order to examine the mutual influence of transcription speed and refolding kinetics on the population of intermediate riboswitch conformations we performed simulations based on a kinetic Markov-model (Prinz et al., 2011) using the experimentally determined refolding and transcription rates of 20 nt/s. This value is based on the apparent rates of transcription determined in the transcription assays for *B. subtilis* polymerase but taking into account the cellular concentrations of nucleotides, that are approx. 10 times higher than those used in the in vitro transcription reactions (Buckstein et al., 2008) (Figure 5). In the absence of ligand, there is no major change as the intermediate conformations occur in a linear succession. In both cases, with and without transcriptional pausing, RNA synthesis is fast enough to allow for formation of the functional on-state apo-form conformation P[AT] (Figure 5b–e). Refolding to the thermodynamic most stable functional off-state conformation [PA][TH] in its apo-form only occurs after the polymerase has passed the regulatory point of decision. In presence of the ligand the conformational space is described as a branched succession of intermediates (Figure 5a). Without pausing of the transcription machinery, riboswitch synthesis is fast compared to its own refolding reactions from apo- to holo-conformations. Therefore, the GSW does not reach a substantial population of conformations with the formed terminator [TH] and the polymerase escapes termination (Figure 5f,g). In contrast, pausing of transcription at pause site 1 opens a time window that allows for refolding of the riboswitch from the apo- to the holo-form. Consequently, the [PA] conformation is formed which is stable over the time window of regulation. In continuation of transcription strand T is synthesized but because it can no longer rapidly associate with strand A to form an anti-terminator conformation, it will form the terminator hairpin conformation [TH] with strand H, as the elongation of the riboswitch by the polymerase occurs with rates not allowing dissociation of the [PA] helix. Therefore, a reduced rate of transcription after formation of the aptamer nucleotides of the riboswitch due to pausing at PS1 in conjunction with a comparative slow conformational transition from [PA]T to P[AT] results in higher population of off-states during synthesis before the polymerase leaves the point of decision (Figure 5h,i).

**Figure 6.**
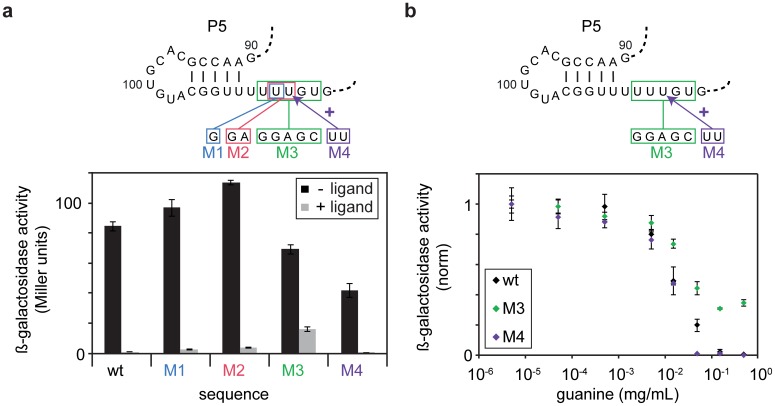
In vivo Pause site characterisation. (**a**) Regulation of ß-galactosidase reporter gene expression by wt-GSW and pause site 1 mutants. Nucleotide exchanges or insertion of residues to generate the PS1 mutants M1–M4 are indicated. Enzyme activity for cells grown in absence (*black bars*) or presence of 0.5 mg mL^−1^ guanine (*grey bars*), respectively; the dynamic range corresponds to the ratio of enzyme activity in absence and presence of ligand. (**b**) Dose-dependent repression of ß-galactosidase expression for wt GSW (*black*), M3 (*green*) and M4 (*purple*). Nucleotide exchanges to generate the PS1 mutants M3 and M4 are indicated. Cultures were grown with increasing concentrations of guanine. Deletion of PS1 reduces riboswitch efficiency, whereas it is increased in case of elongation of PS1. However, both mutations do not significantly alter the half maximal effective concentration EC50. **DOI:** http://dx.doi.org/10.7554/eLife.21297.015

In order to validate the derived model, we tested whether the pause site PS1 could further increase the time for ligand binding to the aptamer domain and shift the riboswitch towards off-state function in an in vivo reporter assay. Successive disruptive mutations (M1-M3) of PS1 correlated with the loss of regulation in vivo (Figure 6a), and the addition of further U residues to PS1 (M4) increased riboswitch efficiency. Monitoring the dose-dependence of the wild-type riboswitch compared to the M3 and M4 mutants indicated that the loss of pause site integrity reduced the switching efficiency, whereas extended pausing of the RNA polymerase led to increased repression of reporter gene expression (Figure 6b). Hence, pausing of the polymerase at PS1 temporarily decouples the continuous synthesis of the downstream antiterminator sequence, assists in folding of the aptamer domain and allows for sufficient time for the ligand to bind at the structural decision point.

## Discussion

Single molecule force extension experiments showed that the structurally similar aptamer domain of the adenine-sensing riboswitch folds co-transcriptional in a hierarchical manner and that the closure of the PA is the last step in accommodation of the ligand in the holo-form (Greenleaf et al., 2008). These findings and our proposed model are consistent as both conclude that the formation of the base-paired PA-stem is the committed-step in the off-pathway. Our experiments complement the previous data, however, by investigating the full-length riboswitch, which is important to map out the coexistence of ligand-dependent and ligand-independent functional off-states (Helmling et al., 2017). For this, we show that one of the two proposed functionally important conformational states of transcriptional riboswitches, the antiterminator state, represents a metastable conformation. The structural analysis using static and dynamic experiments performed close to the thermodynamically equilibrium indicate that an additional kinetic component is needed for adoption of the antiterminator conformation during transcription to allow full biological function of the riboswitch. Pausing during RNA synthesis and the inherent influence on the conformation of the nascent RNA chain is likely required for formation of the antiterminator. Without this additional kinetic component, the terminator would always form and transcription of the adjacent genes would never occur. This is reminiscent of the notion that transcriptional riboswitches are kinetically driven, as here the folding of the RNA has to compete with the speed of the transcribing polymerase. For the FMN switch from *Bacillus subtilis,* it was shown that FMN binding and subsequent folding into the terminator conformation requires pausing of the bacterial polymerase (Wickiser et al., 2005). Unexpectedly, our simulations show that without pausing, the antiterminator conformation is always adopted, independently of ligand concentration. Without pausing, effective regulation could not be achieved by the riboswitch because gene expression could not be turned off. Under these conditions, the thermodynamically favored terminator conformation is not adopted. The presence of the multiple pausing events between the formation of the binding competent aptamer and the transcription of the switching T-strand allow the terminator to form and fulfill its regulatory purpose. In addition we showed that the polymerases of *B. subtilis* and *E. coli* utilize different pausing sequences (e.g. RNA95 and RNA90) and therefore differently affect conformational change in the synthesized RNA. Thus, the effective biotechnological application of transcription-regulating riboswitches in heterologous organisms may require a match between polymerase pausing sequences and the conformational space of the riboswitch. Our work significantly extends our understanding of the previously introduced concept of kinetic control of riboswitches, and reveals the importance of matching discontinuous transcription rates to folding rates of transient conformations in order to facilitate RNA-based regulation of gene expression.

## Materials and methods

### RNA constructs

RNA constructs GSW^PATH^, GSW^PAT^, GSW^PA^, dGSW and FSW were synthesised by in vitro transcription using T7 polymerase and purified as described (Fürtig et al., 2003). Antisense oligonucleotide S2^trans^ was purchased from Thermo Fisher (Epsom, Great Britain), 7-deazaG147 modified S3^trans^ was obtained from IBA (Göttingen, Germany).

### NMR-spectroscopic experiments

Unless stated otherwise, all NMR experiments were performed in NMR buffer (2 mM magnesium chloride, 50 mM potassium chloride, 25 mM potassium phosphate, pH 6.2) containing 10% or 5% D_2_O (static or kinetic experiments) on Bruker spectrometers (Rheinstetten, Germany) and analysed with Topspin (Bruker, Rheinstetten, Germany). Hypoxanthine was used as the better soluble ligand. Real-time NMR experiments were performed at 283 K utilising a rapid sample mixing device (Mok et al., 2003). Selectively ^15^N-U and ^15^N-G,U labelled GSW^PA^ and non-labelled antisense oligonucleotides T^trans^ and H^trans^ were used in combination with a pulse sequence containing an x-filter element to improve the spectral resolution. The RNA concentrations were chosen to be 150 µM after the mixing event; RNAs GSW^PA^, T^trans^ and H^trans^ were in a 1:1(:1) ratio, 4 equivalents of hypoxanthine were added. Ligand binding and conformational rearrangements were monitored on the resolved imino proton resonances reporting on the corresponding interactions. Association of GSW^PA^ and T^trans^ was performed in a 1:2 ratio in 50 mM potassium chloride, 25 mM potassium phosphate, pH 6.2.

### ITC

ITC measurements were performed with a Microcal VP ITC (Northampton, MA USA) at 283 K. A 217 µM solution of hypoxanthine as ligand was titrated to a 15 µM solution of RNA using 20–42 injections. Buffer conditions were 2 mM magnesium chloride, 50 mM potassium chloride, 25 mM potassium phosphate, pH 6.2. Data were analysed with the Origin ITC software (OriginLab, Northampton, MA USA) assuming a single binding site.

### Simulations

Kinetic Markovian simulations were performed in order to evaluate the switching behaviour of the guanine sensing riboswitch under different transcription synthesis velocities. The model assumes N discrete states each representing a distinct length and associated conformation of the RNA chain. The kinetics between the states is described by first order rate equations with a single rate constant *k*_ij_ for the inter-state transition i to j. For the synthesis steps in the evaluated models, a transcription rate of 20 nt s^−1^ was assumed. The rates connecting conformational states by refolding reactions were employed as determined here from the real-time NMR measurements. Two sets of simulations were performed, in the first set the transcription rate was adjusted to the experimental value for transcription without any pausing events, while the second set simulated a pausing event between states PA/^lig^PA and PAT/^lig^PAT. Both sets of simulations were performed for absence and presence of cognate ligand. For the pausing events the transcription rate was set to the reduced transcription rate determined in the transcription assays. Thereby, the reduction of the transcription rate over the pause site corresponds to a factor of ≈70 in the simulations.

### Expression and purification of the *B. subtilis* RNA-Polymerase

#### Cell cultures

The *B. subtilis* MH5636 strain was grown in Bacillus growth medium (5 g tryptone, 10 g yeast extract, 5 g NaCl, and 10 g dextrose per liter) at 37°C with shaking at 200 rpm to a OD_600_ of 1.0–1.2. The cells were harvested by centrifugation with 5000 rpm for 15 min at 4°C. The pellet was frozen at −80°C.

#### French press

Cells (60 grams) were thawed on ice and resuspended in 120 ml Lysis buffer (50 mM Tris-HCl pH 8.0, 2 mM EDTA, 230 mM NaCl, 5% glycerol, 1 mM ß-mercaptoethanol, 1 mM DTT, 1 mM phenylmethylsulfonyl fluoride, 1 ml of a protease inhibitor cocktail (31.2 mg/L benzamide, 0.5 mg/L chymostatin, 0.5 mg/L leupeptin, 0.1 mg/L aprotonin, and 1 mg/L antipain). After cells were fully resuspended, 0.1 mg/mL lysozyme was added to the cells on ice. The cell suspension was lysed in the cold using a French Press at 1000 PSI. The lysate was then centrifuged for 15 min with 12000 rpm at 4°C. The resulting supernatant was transferred to new centrifuge tubes and again centrifuged for 15 min with 12000 rpm at 4°C.

#### Polyethyleneimine (PEI) and ammonium sulfate precipitation

In the cold, the supernatant was transferred to a 250 mL beaker and 8% PEI was added slowly (0.5 ml added every 5 min) with constant stirring to a final concentration of 0.6%. After the last PEI addition, the solution was stirred for an additional 30 min and then centrifuged for 15 min at 12000 rpm at 4°C.

The supernatant was removed and the PEI pellet was washed with 200 mL TGEZ (10 mM Tris-HCl pH 8.0, 0.1 mM EDTA, 5 µM ZnCl_2_, 5% glycerol, 5 mM ß-mercapthothanol) + 0.4 M NH_4_Cl. The pellet was properly dispensed in the buffer. The suspension was centrifuged for 15 min with 12000 rpm at 4°C.

The pellet was resuspended in 200 mL TGEZ +1 M NH_4_Cl for protein elution. The pellet was completely resuspended in the buffer and then the solution was centrifuged for 15 min with 12000 rpm at 4°C.

The supernatant was transferred to a prechilled beaker. Ammonium sulfate was gradually added to a final concentration of 37% with constant stirring. After the last addition, the solution was stirred overnight at 4°C. In the morning, the solution was centrifuged for 15 min at 12000 rpm at 4°C.

#### Ni^2+^ chromatography using His-Trap column

The pellet was resuspended in Loading buffer (25 mM Tris-HCl pH 8.0, 2 mM EDTA, 230 mM NaCl, 5% glycerol, 10 mM imidazole, and 5 mM ß-mercaptoethanol) and centrifuged at 12000 rpm for 15 min at 4°C. The resulting supernatant was then filtered and loaded onto a His-trap column equilibrated in the same buffer at a flow rate of 1 ml/min. After washing with ten column volumes, the protein was eluted using a gradient of 0.01 M to 0.5 M imidazole in the same buffer over 30 min, then from 0.5 M to 0.75 M over 10 min, and then 1 M imidazole. 2 ml fractions were collected.

The fractions were analyzed by 4–12% gradient PAGE and Bradford assay. The RNAP was eluted in fractions 7–23, which corresponded to buffer with 200–850 mM imidazole. The combined fractions were dialyzed overnight in TGEZ +100 mM NaCl +5 mM DTT.

#### Heparin Hi-Trap column

The dialyzed solution was filtered and then loaded onto the heparin column equilibrated in the same buffer. After washing with the same buffer for 10 column volumes, the protein was then eluted in the same buffer using a gradient to 1 M NaCl.

Protein-containing fractions were pooled and dialyzed into storage buffer (10 mM Tris-HCl pH 8.0, 25% glycerol, 0.1 mM EDTA, 100 mM NaCl, 20 µM ZnCl_2_, 1 mM MgCl_2_, and 10 mM DTT) overnight. The total yield of the core enzyme (ε_260_ = 210025 M^−1^.cm^−1^) was 5 mL of 0.83 mg/mL, 2.5 µM solution.

### Purification of *B. subtilis* SigA

#### Cell culture

*E. coli* Rosetta 2 DE3 cells were transformed with plasmid pT7_Bs_sigma_A and selected on LB agar plates with antibiotics (20 µg/mL Kanamycin and 20 µg/mL Chloramphenicol) at 37°C. From this plate, a single transformant was grown at 37°C in 5 ml LB medium with antibiotics. This culture was then used to inoculate 2 liters of LB with antibiotics. The cultures were grown with shaking at 37°C to an OD_600_ of 0.5. The cultures were then shifted to 16°C and the overexpression of SigA (43 kDa) was induced by addition of 1 mM IPTG. Cultures were maintained at 16°C with shaking overnight. The cells were then harvested by centrifugation at 5000 rpm for 15 min at 4 C. The 8.5 g pellet was frozen at −80°C.

#### Sonication

Cells were thawed on ice and resuspended in 35 ml SigA Lysis buffer (50 mM Tris-HCl pH 8.0, 2 mM EDTA, 233 mM NaCl, 5% glycerol, 5 mM ß-mercaptoethanol, 1 mM phenylmethylsulfonyl fluoride, and 350 ml of a protease inhibitor cocktail (31.2 mg/L benzamide, 0.5 mg/L chymostatin, 0.5 mg/L leupeptin, 0.1 mg/L aprotonin, and 1 mg/L antipain). The resuspended cells were transferred to a prechilled beaker and lysosyme was added (0.130 mg/mL) and the cells were incubated on ice for 15 min. Sodium deoxycholate was then added to 0.05% final and incubated on ice for an additional 15 min. Cells were then subjected to sonication at output 6 with a 20% duty cycle for 30 min. The sonicated lysate was then centrifuged for 15 min with 12000 rpm at 4°C.

#### His-Trap purification

The SigA-containing cell lysate was filtered and then loaded onto a His-Trap column equilibrated in the same buffer plus 10 mM imidazole. After washing with ten column volumes, the protein was eluted using a gradient to the same buffer containing 1 M imidazole.

The protein-containing fractions were pooled and analyzed by 20% SDS-PAGE. As the fractions contained pure SigA, they were dialyzed into SigA Storage Buffer (10 mM Tris-HCl pH 8.0, 30% glycerol, 0.1 mM EDTA, 100 mM NaCl, 20 µM ZnCl_2_, 1 mM MgCl_2_, and 0.1 mM DTT)) overnight. The resulting protein solution contained 5 mL of 25 mg/mL or 590 µM (ε_260_ = 24410 M^−1^.cm^−1^).

### Time-resolved transcription assay

The DNA template used for time resolved transcription analysis was composed of either a modified native promoter (*B. subtilis* wt promoter with A-35T, T-35G, G-33A mutations) or the strong lambda pR promoter, followed by the GSW-coding sequence from the *B. subtilis* genome (GSW, Supplementary Information Supplementary Sequence 1). For halted complex formation (HC), 25 nM DNA template and 40 nM RNAP holoenzyme (expression and purification shown in SI) were incubated in transcription buffer (20 mM Tris-OAc pH 7.7, 40 mM KOAc, 5 mM Mg(OAc)_2_, and 5 mM ß-mercaptoethanol) with 10 µM ATP and UTP, 7.5 µM GTP, and 150 µM ApA (for the *B. subtilis* GSW promoter) or ApU dinucleotide (for the lambda pR promoter). For transcriptions using the E.cRNAP, 10 µCi GTP* were used whereas transcriptions using the BsRNAP used 20 µCi GTP*. The HC of EcRNAP seem to be more stable than the ones with BsRNAP. Additional GTP* and therefore increased incorporation of ^32^P-labeled nucleotides into the nascent RNA, made more of the fewer transcribed RNAs visible on the PAGEs. Ligand (Hypoxanthine, Sigma Aldrich) was added to a final concentration of 200 µM. Purified rNTPs were obtained from Promega and dinucleotides were obtained from TriLink BioTechnologies. Synchronized transcription was re-started by adjustment of the ATP, CTP, and UTP concentrations to 150 µM and the GTP concentration to 10 µM, and the addition of heparin to a final concentration of 100 µg/mL. Transcription was stopped at different time points by the addition of an equal volume of 2X STOP buffer (100 mM Tris-HCl pH 8.3, 100 mM Boric acid, 40 mM EDTA, 7 M urea). For determination of the transcription rates at high NTP-concentration, the NTP-concentration was raised to 1 mM. After heating to 95°C for 2 min, the reactions were analyzed by 8% PAGE. The gels of the time-resolved transcription assays show several expected RNA signals (Figure 4): pausing 1 fragment (PS1, 110 nt), pausing 2 fragment (PS2, 141 nt), terminated transcript (Gsw^[PA][TH]^, 164 nt), and full–length transcript (FL, 214 nt) as well as two additional pausing events at 77 nt (RNA77) and 95 nt (RNA95) for *E. coli* RNAP mediated transcriptions and 77 nt (RNA77) and 90 nt (RNA90) for *B. subtilis* RNAP mediated transcription. The positions of the RNA species were as estimated by comparison on high-resolution denaturing polyacrylamide gels of transcripts to RNA sequence ladders generated using 3' deoxyNTPs. To do this, halted ECs were first generated as described above. To generate sequence ladders, transcription was restarted by addition of ATP, CTP, GTP, and UTP at 250 µM each, and one 3′-deoxyNTP in each of 4 separate reactions at 240 µM dGTP, 300 µM dATP, 350 µM dCTP, or 400 µM dUTP, followed by addition of heparin at 100 µg/ml. After incubation for 10 min at 37°C, samples were mixed with an equal volume of 2X STOP buffer before being electrophoresed next to transcription products obtained by regular in vitro transcription as described above. To quantitate the relative amounts of the different RNA species, the signal intensities were plotted against time and analyzed using Lab Figure 1D software (INTAS, Göttingen, Germany) and Image Quant 5.2 (GE, Freiburg, Germany). The mean apparent transcription rates *k*app for FL, Gsw^[PA][TH]^, PS2 and PS1 were derived from the exponential plots as shown in by Ederth et al. (2006)). The signal intensities of RNA95, RNA90 and RNA77 were plotted against time and revealed that these fragments were transcribed with apparent first-order kinetics. The *kapp* was derived similar as previously described, by determining the average appearance rate for the first values. Knowing the length of the RNA fragment, the rates in nt s-1 were then calculated from the *k*app (Supplementary file 1). Determination of *t*_1/2_, τ, and *k*p were performed according to Landick et al. (1996).

### Regulation of ß-galactosidase reporter gene expression by the wildtype GSW (wt) and pause site mutants (M1 – M4)

For reporter gene assays, the 5’-UTR of the *B. subtilis xpt-pbuX* operon (encompassing nt −120 to +196 relative to the transcription start site) was fused directly upstream of the *lacZ* reporter gene of plasmid pDG268 (Antoniewski et al., 1990). Pause site mutants M1 – M4 were introduced by PCR mutagenesis. The resulting plasmids were integrated into the *amyE* locus of *B. subtilis* 168. The respective strains were grown in modified CSK minimal medium (Martin-Verstraete et al., 1990) with increasing concentration of guanine. After 5 hr growth, β-galactosidase activity was determined as follows: 100 µL culture aliquots were incubated at 301 K in buffer (60 mM Na_2_HPO_4_, 40 mM NaH_2_PO_4_, 10 mM KCl, 1 mM MgCl_2_ and 50 mM 2-mercaptoethanol) with lysozyme and Triton-X treatment. 200 μL of *o*-nitrophenyl-β-D-galactoside (4 mg mL^−1^) was added, and the sample was incubated at 301 K. The reaction was stopped by addition of 0.5 mL of 1 M Na_2_CO_3_ upon appearance of a yellowish colouration. The time for colour change was noted and absorbance at 420 nm and 550 nm was measured. ß-galactosidase activity was calculated in Miller units, i.e. 1000 x [*A*_420_ – 1.75 x *A*_550_] / [*t* x *V* x *A*_600_]. Three independent cultures were measured in parallel. The measurements were repeated twice.

## Additional files

Supplementary file 1. This file contains rates obtained by Kinetic NMR experiments, the exact nucleotide sequence of the DNA template used in transcriptional assays, as well transcription rates and pause site characteristics. **DOI:** http://dx.doi.org/10.7554/eLife.21297.016
