# Supplementary material for: Pausing guides RNA folding to populate transiently stable RNA structures for riboswitch-based transcription regulation
Source: eLife. 2017 May 25;6:e21297. doi: 10.7554/eLife.21297 (PMC5459577; doi:10.7554/eLife.21297)
Supplement: Supplementary file 1. — DOI: http://dx.doi.org/10.7554/eLife.21297.016 [file elife-21297-supp1.docx]

**Supplementary Information**

**Pausing guides RNA folding to populate transiently stable RNA structures for riboswitch-based transcription regulation**

Hannah Steinert^1+^, Florian Sochor^1+^, Anna Wacker^1^, Janina Buck^1^, Christina Helmling^1^, Fabian Hiller^1^, Sara Keyhani^1^, Jonas Noeske^1^, Steffen Grimm^1^, Martin M. Rudolph^2^, Heiko Keller^3^, Rachel Anne Mooney, Robert Landick, Beatrix Suess^2^, Boris Fürtig^1,*^, Jens Wöhnert^3,^*, and Harald Schwalbe^1,^*

^1^Center for Biomolecular Magnetic Resonance, Institute of Organic Chemistry and Chemical Biology, Johann Wolfgang Goethe-University Frankfurt am Main, Max-von-Laue-Strasse 7, 60438 Frankfurt am Main/ Germany

^2^Department of Biology, Technical University Darmstadt, Schnittspahnstraße 10, 64287 Darmstadt/ Germany

^3^Center for Biomolecular Magnetic Resonance, Institute of Molecular Biosciences, Johann Wolfgang Goethe-University Frankfurt am Main, Max-von-Laue-Strasse 9, 60438 Frankfurt am Main/ Germany

^4^1550 Linden Drive, Microbial Sciences Building, University of Wisconsin – Madison, Madison WI 53706

**Kinetic NMR experiments**

Letters b-h refer to the scheme in Figure 3a in the main article.

**Supplementary Table 1: Annealing of GSW^PA^ and T^trans^.** Kinetic experiment b.

|  |  | *k* |
| --- | --- | --- |
| structure motif | reporter signal | (10^-3^ s^-1^) |
| PA dissociation | U17 | 6 ± 2 |
|  | G79 | 17 ± 8 |
| AT formation | G79 & G131 | 7 ± 2 |
|  | U81 | 6 ± 2 |

**Supplementary Table 2: Formation of the terminator helix T^trans^–H^trans^ from GSW^PA^-T^trans^ in the absence of ligand.** Kinetic experiment c.

|  |  | *k*_1_ | *k*_2_ |
| --- | --- | --- | --- |
| structure motif | reporter signal | (10^-3^ s^-1^) | (10^-4^ s^-1^) |
| PA formation | U17 | 0.40 ± 0.05 | 0.7 ± 0.5 |
|  | G79 | 0.43 ± 0.05 | 0.7 ± 0.3 |
| AT dissociation | G79 | 0.48 ± 0.06 | 1.0 ± 0.2 |
|  | G82 | 0.51 ± 0.08 | 1.3 ± 0.2 |
|  | U130 | 0.42 ± 0.05 | 0.7 ± 0.3 |
|  | G131 | 0.42 ± 0.04 | 0.7 ± 0.3 |
|  | U133 | 0.46 ± 0.05 | 0.9 ± 0.3 |
| TH formation | U129 | 0.81 ± 0.07 | 1.5 ± 0.07 |
|  | U130 | 0.61 ± 0.02 | 1.21 ± 0.04 |
|  | G131 | 0.79 ± 0.2 | 1.5 ± 0.2 |
|  | U133 | 0.56 ± 0.07 | 1.2 ± 0.2 |
|  | G144 | 0.41 ± 0.06 | 0.7 ± 0.4 |
|  | G146 | 0.65 ± 0.04 | 1.42 ± 0.06 |
|  | G147 | 0.62 ± 0.05 | 1.31 ± 0.09 |

**Supplementary Table 3: Ligand binding to GSW^PA^ in complex with T^trans^.** Kinetic experiment d.

|  |  | *k* |
| --- | --- | --- |
| structure motif | reporter signal | (10^-3^ s^-1^) |
| ligand binding | U47 | 0.88 ± 0.09 |
|  | U49 | 0.92 ± 0.08 |
|  | U51 | 0.96 ± 0.09 |
| PA formation | U17 | 0.8 ± 0.2 |
|  | G79 | 1.1 ± 0.3 |
| AT dissociation | G79 & G131 | 0.83 ± 0.03 |
|  | U81 | 0.92 ± 0.05 |
|  | G82 | 0.85 ± 0.08 |
|  | U129 | 0.9 ± 0.2 |
|  | U130 | 0.83 ± 0.06 |
|  | U133 | 0.78 ± 0.05 |

**Supplementary Table 4: Formation of the terminator helix T^trans^–H^trans^ from GSW^PA^-T^trans^ in presence of the ligand.** Kinetic experiment e.

|  |  | *k*_1_ | *k*_2_ |
| --- | --- | --- | --- |
| structure motif | reporter signal | (10^-2^ s^-1^) | (10^-3^ s^-1^) |
| ligand binding | U47 | 2.6± 0.8 | 1.0 ± 0.2 |
|  | U49 | 2.3 ± 0.5 | 1.1 ± 0.2 |
| PA formation | U17 | 1.1 ± 0.4 | 0.9 ± 0.4 |
|  | G79 | 0.5 ± 0.2 | 0.6 ± 0.6 |
| AT dissociation | G79 |  | 2.2 ± 0.3 |
|  | U81 |  | 1.8 ± 0.2 |
|  | G82 |  | 2.2 ± 0.4 |
|  | G131 |  | 1.7 ± 0.3 |
|  | U133 |  | 2.1 ± 0.4 |
| TH formation | U129 | 1.6 ± 0.3 | 1.3 ± 0.2 |
|  | U130 | 2.0 ± 0.2 | 1.1 ± 0.1 |
|  | G131 | 3 ± 2 | 1.4 ± 0.4 |
|  | U133 | 4 ± 2 | 1.0 ± 0.4 |
|  | G144 | 2 ± 1 | 1.2± 0.5 |
|  | G146 | 2.1 ± 0.4 | 1.4 ± 0.2 |
|  | G147 | 2.6 ± 0.6 | 1.8 ± 0.3 |
|  | G152 | 1.7 ± 0.5 | 1.6 ± 0.4 |

**Supplementary Table 5: Ligand binding to GSW^PA^.** Kinetic experiment f.

|  |  | *k*_1_ | *k*_2_ |
| --- | --- | --- | --- |
| structure motif | reporter signal | (10^-1^ s^-1^) | (10^-2^ s^-1^) |
| ligand binding | U47, U49 & U51 | 2.1 ± 0.5 | 2 ± 1 |

**Supplementary Table 6: Ligand binding to GSW^PA^ in the presence of the terminator helix T^trans^–H^trans^.** Kinetic experiment g.

|  |  | *k*_1_ | *k*_2_ |
| --- | --- | --- | --- |
| structure motif | reporter signal | (10^-1^ s^-1^) | (10^-2^ s^-1^) |
| ligand binding | U47 & U49 | 3 ± 2 | 2 ± 1 |

**Supplementary Table 7: Formation of the terminator helix T^trans^–H^trans^ from single strands.** Kinetic experiment h.

|  |  | *k*_1_ | *k*_2_ |
| --- | --- | --- | --- |
| structure motif | reporter signal | (10^-1^ s^-1^) | (10^-4^ s^-1^) |
| TH formation | U125 | 1.3 ± 0.2 | 1.4 ± 0.3 |
|  | G127 | 2.1 ± 0.2 | 1.2 ± 0.2 |
|  | U129 | 1.10 ± 0.08 | 1.09 ± 0.08 |
|  | U130 | 1.14 ± 0.07 | 0.95 ± 0.06 |
|  | G131 | 1.3 ± 0.2 | 0.7 ± 0.2 |
|  | U133 | 1.0 ± 0.2 | 1.3 ± 0.3 |
|  | G142 | 3.7 ± 0.7 | 1.7 ± 0.3 |
|  | G144 | 0.8 ± 0.2 | 1.2 ± 0.2 |
|  | G146* | 1.34 ± 0.06 | 0.75 ± 0.07 |
|  | G147 | 1.05 ± 0.07 | 1.1 ± 0.1 |
|  | G152 | 1.2 ± 0.1 | 1.5 ± 0.2 |
|  | G153 | 2.1 ± 0.2 | 0.8 ± 0.2 |

**Transcription rate determination and pause site analysis**

For the pause site analysis, we used the following construct consisting of a promoter region (bold), a synchronizer (underlined) and the coding sequence of the GSW (italic) including the first 23 base pairs of the adjacent gene.

Supplementary Sequence 1: Nucleotide sequence of GSWsync

**TTATCAAAAAGAGTATTGACTTAAAGTCTAACCTATAGGATACTTACAGCC**AGCAGCAGCAGCAGCAGC*AATATAATAGGAACACTCATATAATCGCGTGGATATGGCACGCAAGTTTCTACCGGGCACCGTAAATGTCCGACTATGGGTGAGCAATGGAACCGCACGTGTACGGTTTTTTGTGATATCAGCATTGCTTGCTCTTTATTTGAGCGGGCAATGCTTTTTTTATTCTCATAACGGAGGTAGACAGGATGGAAGCACTGAAACGGAAAAT*

**Supplementary Table 8: Transcription rates and pause site characteristics of the RNA fragments obtained from the time-resolved transcription assay.**

Average time of appearance (of three experiments) of the RNA-fragments as used to estimate the elongation rate for the *E. coli* RNAP transcription in the absence of ligand (Ederth J, Mooney RA, Osaksson LA, Landick R, 2005) . The transcriptions were measured at low NTP-concentration as an increase especially for GTP resulted in the signal loss for the pause RNA-fragments.

|  | t app (s) | Error (s) | Length (nt) | kapp (nt/s) | Error (nt/s) |
| --- | --- | --- | --- | --- | --- |
| FL | 108.1 | 27.2 | 214.0 | 2.0 | 0.5 |
| Gsw^PATH^ | 119.7 | 25.2 | 164.0 | 1.4 | 0.3 |
| PS2 | 22.1 | 8.0 | 141.0 | 6.4 | 2.3 |
| PS1 | 27.4 | 9.4 | 110.0 | 4.0 | 1.4 |
| RNA95 | 23.9 | 2.3 | 95.0 | 4.0 | 0.4 |
| RNA77 | 44.8 | 5.6 | 77.0 | 1.7 | 0.2 |

Average time of appearance (of three experiments) of the RNA-fragments as used to estimate the elongation rate for the *E. coli* RNAP transcription in the presence of ligand. The transcriptions were measured at low NTP-concentration as an increase especially for GTP resulted in the signal loss for the pause RNA-fragments.

|  | t app (s) | Error (s) | Length (nt) | kapp (nt/s) | Error (nt/s) |
| --- | --- | --- | --- | --- | --- |
| FL | 275.8 | 123.8 | 214.0 | 0.8 | 0.3 |
| Gsw^PATH^ | 256.1 | 83.8 | 164.0 | 0.6 | 0.2 |
| PS2 | 72.9 | 42.9 | 141.0 | 1.9 | 1.1 |
| PS1 | 84.6 | 39.7 | 110.0 | 1.3 | 0.6 |
| RNA95 | 22.5 | 1.8 | 95.0 | 4.2 | 0.3 |
| RNA77 | 37.5 | 2.1 | 77.0 | 2.1 | 0.1 |

Average time of appearance (of three experiments) of the RNA-fragments as used to estimate the elongation rate for the *B. subtilis* RNAP transcription in the absence of ligand. The transcriptions were measured at low NTP-concentration as an increase especially for GTP resulted in the signal loss for the pause RNA-fragments.

|  | t app (s) | Error (s) | Length (nt) | kapp (nt/s) | Error (nt/s) |
| --- | --- | --- | --- | --- | --- |
| FL | 384.8 | 81.2 | 214.0 | 0.6 | 0.1 |
| Gsw^PATH^ | 441.7 | 59.5 | 164.0 | 0.4 | 0.1 |
| PS2 | 3.0 | 3.4 | 141.0 | 47.0 | 53.3 |
| PS1 | 36.7 | 10.8 | 110.0 | 3.0 | 0.9 |
| RNA90 | 12.4 | 1.8 | 90.0 | 7.3 | 1.0 |
| RNA77 | 13.2 | 1.5 | 77.0 | 5.8 | 0.7 |

Average time of appearance (of three experiments) of the RNA-fragments as used to estimate the elongation rate for the *B. subtilis* RNAP transcription in the presence of ligand. The transcriptions were measured at low NTP-concentration as an increase especially for GTP resulted in the signal loss for the pause RNA-fragments.

|  | t app (s) | Error (s) | Length (nt) | kapp (nt/s) | Error (nt/s) |
| --- | --- | --- | --- | --- | --- |
| FL | 253.7 | 86.1 | 214.0 | 0.8 | 0.3 |
| Gsw^PATH^ | 775.9 | 89.4 | 164.0 | 0.2 | 0.0 |
| PS2 | 6.7 | 4.0 | 141.0 | 21.0 | 12.6 |
| PS1 | 61.4 | 15.9 | 110.0 | 1.8 | 0.5 |
| RNA90 | 7.8 | 1.2 | 90.0 | 11.5 | 1.7 |
| RNA77 | 9.6 | 0.1 | 77.0 | 8.0 | 0.1 |

Pausing parameters for the identified pause sites of the transcription using the *E. coli* RNAP in the absence of ligand.

|  | τ (s) | Error (s) | kap (s^-1^) | Error (s^-1^) | t1/2 (s) | Error (s) |
| --- | --- | --- | --- | --- | --- | --- |
| PS2 | 165.0 | 55.3 | 0.006 | 0.018 | 114.4 | 38.3 |
| PS1 | 238.7 | 391.5 | 0.004 | 0.003 | 165.5 | 271.4 |
| RNA95 | 129.2 | 40.4 | 0.008 | 0.025 | 89.6 | 28.0 |
| RNA77 | 3.0 | - | 0.333 | - | 2.1 | - |

Pausing parameters for the identified pause sites of the transcription using the *E. coli* RNAP in the presence of ligand.

|  | τ (s) | Error (s) | kap (s^-1^) | Error (s^-1^) | t1/2 (s) | Error (s) |
| --- | --- | --- | --- | --- | --- | --- |
| PS2 | 99.7 | 25.3 | 0.010 | 0.040 | 69.1 | 17.5 |
| PS1 | 291.9 | 110.9 | 0.003 | 0.009 | 202.3 | 76.9 |
| RNA95 | 87.1 | 25.4 | 0.011 | 0.039 | 60.4 | 17.6 |
| RNA77 | 42.3 | 12.0 | 0.024 | 0.083 | 29.3 | 8.3 |

Pausing parameters for the identified pause sites of the transcription using the *B. subtilis* RNAP in the absence of ligand.

|  | τ (s) | Error (s) | kap (s^-1^) | Error (s^-1^) | t1/2 (s) | Error (s) |
| --- | --- | --- | --- | --- | --- | --- |
| PS2 | 74.3 | 9.9 | 0.013 | 0.101 | 51.5 | 6.9 |
| PS1 | 158.5 | 65.4 | 0.006 | 0.015 | 109.9 | 45.3 |
| RNA95 | 55.2 | 11.0 | 0.018 | 0.091 | 38.3 | 7.6 |
| RNA77 | 78.5 | 9.0 | 0.013 | 0.111 | 54.4 | 6.2 |

Pausing parameters for the identified pause sites of the transcription using the *B. subtilis* RNAP in the presence of ligand.

|  | τ (s) | Error (s) | kap (s^-1^) | Error (s^-1^) | t1/2 (s) | Error (s) |
| --- | --- | --- | --- | --- | --- | --- |
| PS2 | 56.8 | 12.6 | 0.018 | 0.079 | 39.4 | 8.7 |
| PS1 | 95.5 | 32.9 | 0.010 | 0.030 | 66.2 | 22.8 |
| RNA95 | 51.6 | 9.4 | 0.019 | 0.106 | 35.8 | 6.5 |
| RNA77 | 59.6 | 12.3 | 0.017 | 0.081 | 41.3 | 8.5 |

**References:**

1 Gautheret, D. & Lambert, A. Direct RNA motif definition and identification from multiple sequence alignments using secondary structure profiles. *J Mol Biol* **313**, 1003-1011, doi:10.1006/jmbi.2001.5102 (2001).

2 Macke, T. J. *et al.* RNAMotif, an RNA secondary structure definition and search algorithm. *Nucleic acids research* **29**, 4724-4735 (2001).

3 Davenport, R. J., Wuite, G. J., Landick, R. & Bustamante, C. Single-molecule study of transcriptional pausing and arrest by E. coli RNA polymerase. *Science (New York, N.Y* **287**, 2497-2500 (2000).

4 Landick, R., Wang, D. & Chan, C. L. Quantitative analysis of transcriptional pausing by Escherichia coli RNA polymerase: his leader pause site as paradigm. *Methods in enzymology* **274**, 334-353 (1996).
