# Supplementary material for: Pausing guides RNA folding to populate transiently stable RNA structures for riboswitch-based transcription regulation
Source: eLife. 2017 May 25;6:e21297. doi: 10.7554/eLife.21297 (PMC5459577; doi:10.7554/eLife.21297)
Supplement: Related texts

# Article commentary: Decision letter

**DOI:** 10.7554/eLife.21297.017

**Affiliations:** University of Toronto, Canada

In the interests of transparency, eLife includes the editorial decision letter and accompanying author responses. A lightly edited version of the letter sent to the authors after peer review is shown, indicating the most substantive concerns; minor comments are not usually included.

Thank you for submitting your article "Pausing guides RNA folding to populate transiently stable RNA structures for riboswitch-based transcription regulation" for consideration by *eLife*. Your article has been favorably evaluated by John Kuriyan (Senior Editor) and four reviewers, one of whom is a member of our Board of Reviewing Editors. The following individual involved in review of your submission has agreed to reveal their identity: Kathleen Hall (Reviewer #4).

The reviewers have discussed the reviews with one another and the Reviewing Editor has drafted this decision to help you prepare a revised submission.

Summary:

The manuscript describes a detailed set of biochemical, biophysical and biological analyses to understand the mechanism used by a guanine riboswitch to control gene expression. The work involves a series of ligand binding studies and folding kinetics that were carried out using truncated riboswitches. This study expands on the original observations that some riboswitches are influenced more by kinetic processes (speed of aptamer folding, rate constant for ligand association, speed of RNA synthesis) than by thermodynamic equilibria. The NMR study in this manuscript is well executed, in particular the characterization of secondary structures of sub-state conformations. One of the reviewer’s comments on the beautiful NMR data and the creative use of imino protons to monitor structure formation. Indeed, all of the reviewers were impressed with the NMR work.

Essential revisions:

1) All of the reviewers were concerned about the fact that the authors did not succeed in establishing how their results go beyond what is currently understood and also that they have not tied their results with those published previously. For example, the authors claim that "Up to now, a high-resolution structural depiction of the concerted process of synthesis and ligand-induced restructuring of the regulatory RNA is missing.". However, there is excellent single-molecule biophysics data from the laboratory of Steven Block for adenine riboswitches (a close relative of guanine riboswitches) (Science [2012], 338:397). This previous paper should be cited and the authors should compare their findings with this prior work. In particular, what is new from this study? Work from the Micura and Block labs that leads to similar conclusions about the importance of kinetics should also be cited. The authors also state that the RNA "…exclusively populates the functional off-state but cannot switch to the on-state, regardless of the presence or absence of ligand.". However, similar claims have been made for FMN riboswitches (Wickiser et al., 2005) and probably others. This brings up an important point: maybe many riboswitches do not switch between the on- and off-states, but choose a folding pathway based on ligand binding early after the full aptamer has been made. The authors should consider this possibility. As indicated above, the mechanism of the guanine-sensing riboswitch described here is very similar to that of the FMN riboswitch, including the number of pause sites and half time of a pause site. However, in this manuscript, little is discussed regarding the mechanism of the FMN riboswitch and there is no comparison between the two riboswitches. The authors need to provide more detailed discussions about the similarity between the two riboswitches and again why this work goes beyond what is currently understood.

2) Related to point 1 is that there is currently no Discussion. Addressing point 1 could be done in a Discussion format although the authors must clarify the novelty in the Introduction as well more clearly.

3) The authors use separate oligonucleotides that act as surrogates for the actual base-paired interactions that occur in a unimolecular riboswitch. It is not clear that these interactions will have kinetic properties that accurately reflect analogous interactions that occur in the natural construct during RNA synthesis. If not, then the author's conclusions (such as those made in the eighth paragraph of the Results) might be flawed.

4) Related somewhat to 3: The associations between PA and T and between T and H are bimolecular reactions in the NMR experiments. However, during co-transcriptional folding, these associations are intramolecular reactions. It is not clear in the manuscript whether the authors have considered differences between these two types of reactions and corrected the NMR-determined associations rates for simulation. Further, the authors did not provide clear descriptions in this manuscript how the simulation was performed, how the pausing was implemented, and what rates were used. For example, t0,1~5s and t0,2~61s were labeled for ligand binding at the aptamer in Figure 5a. These values seem to be calculated from NMR determined k1 and k2 values. Again since the association rate is ligand concentration dependent was it corrected for simulation?

5) One of the major concerns of the current study is how transcription intermediates and pause sites were identified. It is possible that a transcription intermediate gives an aborted product in multi-round transcription, but the appearance of an aborted product in multi-round transcription can be a result of many other factors. The appropriate approach for identifying a transcription intermediate is to measure accumulation in time-resolved single-round transcription assays, such as what the authors have done to confirm pause site 2 (PS2). In this regard, in contrast to PS2, the identity of pause site 1 (PS1) is not clear, as no transcription intermediate at PS1 was detected in the time-resolved single-round transcription assay. However, in this manuscript, PS1 has been a critical intermediate for designing truncated riboswitch constructs, performing kinetic measurements, and simulating co-transcriptional pathways (see below). If PS1 is a true pause site, the authors need to clearly confirm the presence of PS1 and characterize its pausing properties.

6) Similar to the above concern, it is not appropriate to compare aborted products in multi-round transcription reactions to evaluate transcription intermediates between *E. coli* transcription and *B. subtilis* transcription. For example, there are five major products in the *E. coli* transcription, but there are least eight major products shown in the *B. subtilis* transcription. If aborted products were all transcription intermediates, this would suggest there are many more pause sites for the *B. subtilis* polymerase than for the *E. coli* polymerase, which is not likely. To properly evaluate transcription systems, the authors need to perform time-resolved single-round transcription assays and compare differences in transcription intermediates.

7) In the figure for simulation, the authors labeled pausing at PS1 but did not label pausing at PS2. In the manuscript, the authors specifically discussed pausing at PS1 but did not discuss pausing at PS2. The experimental data identified PS2 but did not identify PS1. It is not correct to simply assume the presence of PS1 and ignore PS2. In addition, it is also not clear whether the half time at PS2 was used in the simulation, or whether it was used for PS1.

8) Similar to the above concern, since PS1 was not identified in the in vitro assay, while mutating PS1 sequences do affect in vivo gene expression, it is difficult to evaluate these influences as mutation-induced changes in pausing or other factors, such as folding. The authors need to clearly confirm the presence of PS1 and may also need to characterize pausing properties of these mutations.

9) Regarding the Bacillus and *E. coli* RNAPs: These enzymes are quite different, and they are not considered to be indistinguishable on the basis of their pause kinetics. However, if the authors have new information on their comparative properties, they need to reference it. The authors are using *E. coli* RNAP on a B subtilis riboswitch, which is quite ok, but they need to be clear that they are measuring properties of EC RNAP.

[Editors' note: further revisions were requested prior to acceptance, as described below.]

Thank you for submitting your revised article "Pausing guides RNA folding to populate transiently stable RNA structures for riboswitch-based transcription regulation" for consideration by *eLife*. Your revised article has been favorably evaluated by John Kuriyan as the Senior Editor, Lewis Kay as the Reviewing Editor, and two reviewers. Your paper has been sent for re-review to two of the previous experts who now feel that the work is far improved and suitable for publication at *eLife*.

Both reviewers feel that the work represents an important quantitative advance in the description of transcriptional regulation by riboswitches and that the data is at much higher resolution than earlier studies. One of the reviewers comments about the utility of the simulations of Figure 5 that summarize the importance of ligand binding and pausing to the general function of riboswitches. Additional experiments are not required, but both reviewers do provide suggestions for improvements that you will wish to consider including aspects that would clarify some of the points made in the paper. Please modify your text to address the concerns of the reviewers. We expect to make a decision on this manuscript without needing to re-engage the reviewers.

1) The authors wrote in their response that there are no quantitative models to describe transcriptional regulation by riboswitches. This statement is not correct. While the kinetic model established in the current study represents one of the few quantitative models and has its own merits in advancing the understanding of transcriptional regulation by riboswitches, a similar regulatory model with quantitative kinetic parameters has been previously reported for the FMN riboswitch (Wickiser et al., 2005). The authors need to revise their Introduction to be consistent with the literature.

2) One major concern of the original manuscript was the application of multi-round transcription assays to evaluate transcription intermediates. In the revised manuscript, the authors have performed time-resolved single-round transcription assays to properly establish transcription intermediates. However, despite having these appropriate measurements, it is somewhat surprising that the authors have kept applying the multi-round transcription data to evaluate transcription intermediates (Figure 1E). The authors need to update the text and figure with proper data from their new single-round transcription assays.

3) In the revised manuscript (Results, fifth paragraph), the authors changed the length of one of the transcription intermediates from the original 125 nucleotides to 140 nucleotides. Since this RNA fragment was proposed to represent pause site 2 (PS2), this new length will mark PS2 around nucleotide A150, which is different from PS2 at nucleotide U141 shown in both Figure 1A and Figure 4. The authors need to make sure the location of PS2 is consistent throughout the manuscript.

4) Similar to the above concern, the labels for PS1 and PS2 are confusing in the new Figure 4. For example, PS1 is labeled as 110; however, the RNA band marked as PS1 clearly migrates between the 110 and 123 bands of the sequencing ladder. In addition, the RNA band marked as 141:PS2 migrates right below the 147 band of the sequencing ladder in the Bs RNAP assay. However, in the Ec RNAP assay, there are no apparent RNA bands at similar locations; instead, there is a major RNA band that seems to migrate between the 123 and 147 bands of the sequencing ladder. The authors need to have proper labels for the transcription intermediates in both the Ec RNAP assay and the Bs RNAP assay.

5) In the new supplementary Table 8 (Supplementary file 1), the authors reported transcription rates and pause site characteristics from time-resolved transcription assays. While the majority of the reported values are reasonable, some measurements are quite confusing. For example, the average appearance time of PS2 (141 nts) is shown to be 3.0 seconds in the third table. However, this time value is even less than the average appearance time of the shorter RNA77 (77 nts) fragment (13.2 seconds). In addition, an appearance time of 3.0 seconds is also less than the shortest time point (5 seconds) of the time-resolved assay. Another outlier is the average appearance time of Gsw-PATH, which is shown to be 775.9 seconds in the fourth table. However, it can be seen in Figure 4 that the RNA band for Gsw-PATH starts appearing around 20 – 30 seconds for the Bs RNAP assay. In addition, this appearance time is more than the longest time point (600 seconds) of the time-resolved assay. The authors need to go through the tables and make sure these values are properly reported. If these outliers are authentic, the authors need to comment on them.

6) One other concern of the original manuscript was the lack of details for the co-transcriptional folding simulation. While the authors have provided more details in their revised manuscript, a key aspect of the simulation remains not clear. The authors need to describe in the method how pausing was implemented in the simulation.

# Reply: Author response

**DOI:** 10.7554/eLife.21297.018

*Essential revisions:*

*1) All of the reviewers were concerned about the fact that the authors did not succeed in establishing how their results go beyond what is currently understood and also that they have not tied their results with those published previously. For example, the authors claim that "Up to now, a high-resolution structural depiction of the concerted process of synthesis and ligand-induced restructuring of the regulatory RNA is missing.". However, there is excellent single-molecule biophysics data from the laboratory of Steven Block for adenine riboswitches (a close relative of guanine riboswitches) (Science [2012], 338:397). This previous paper should be cited and the authors should compare their findings with this prior work. In particular, what is new from this study? Work from the Micura and Block labs that leads to similar conclusions about the importance of kinetics should also be cited. The authors also state that the RNA "…exclusively populates the functional off-state but cannot switch to the on-state, regardless of the presence or absence of ligand.". However, similar claims have been made for FMN riboswitches (Wickiser et al., 2005) and probably others.*

We do not agree with the statement that the single-molecule work by Steven Block can be compared in its resolution to NMR structural work where each base-paired nucleotide can be determined. We are not aware that the previous reports would have tried to develop a quantitative model to describe transcriptional regulation, from measurement of ligand binding, RNA refolding from time-resolved NMR and transcription speed. Such studies, even though within reviewing process of this work over the last four years such claims are repeatedly put forward, have not been conducted nor conceptualized before. It is a fundamental difference to put forward a schematic picture or to develop a quantitative kinetic model. Therefore, our work substantially advances the field of transcriptional regulation by riboswitches. From a fundamental point, we find that the regulatory ON state is achieved by a metastable state! This has not been shown before. To highlight this we have extended the Introduction: “*In this contribution, we apply high resolution structural and kinetic techniques to decipher the full co-transcriptional folding pathway, enabling monitoring the behaviour of the decisive structural units during synthesis for the first time.”* Nevertheless, we have included reference to the Block paper and point out the new findings in our study: “In single molecule force extension experiments it could be shown that the structurally similar aptamer domain of the adenine-sensing riboswitch folds co-transcriptional in a hierarchical manner and that the closure of the PA is the last step in accommodation of the ligand in the holo-form (Greenleaf et al., 2008). Although this finding supports our derived model that puts the formation of the base-paired PA-stem to be the committed-step in the off-pathway, the single- molecule force extension experiments are not investigating the full length riboswitch, which is important to map out the coexistence of ligand-dependent and ligand-independent functional OFF-states (Helming et al., 2017).”

*This brings up an important point: maybe many riboswitches do not switch between the on- and off-states, but choose a folding pathway based on ligand binding early after the full aptamer has been made. The authors should consider this possibility. As indicated above, the mechanism of the guanine-sensing riboswitch described here is very similar to that of the FMN riboswitch, including the number of pause sites and half time of a pause site. However, in this manuscript, little is discussed regarding the mechanism of the FMN riboswitch and there is no comparison between the two riboswitches. The authors need to provide more detailed discussions about the similarity between the two riboswitches and again why this work goes beyond what is currently understood.*

In order to address this point and to include a discussion in the light of the new results from transcriptional analysis we have rewritten the entire Discussion section that reads now: “In single molecule force extension experiments it could be shown that the structurally similar aptamer domain of the adenine-sensing riboswitch folds co-transcriptional in a hierarchical manner and that the closure of the PA is the last step in accommodation of the ligand in the holo-form (Greenleaf et al., 2008). […] Our work significantly extends our understanding of the previously introduced concept of kinetic control of riboswitches, and reveals the importance of matching discontinuous transcription rates to folding rates of transient conformations in order to facilitate RNA-based regulation of gene expression.”

*2) Related to point 1 is that there is currently no Discussion. Addressing point 1 could be done in a Discussion format although the authors must clarify the novelty in the Introduction as well more clearly.*

In new single round transcription assays, we show accumulation of several transcriptional intermediates, with pausing and stalling events be present. We were able to do so because the Schwalbe group sent a postdoc to the lab of Landing to conduct such single round transcription in *B. subtilis*. Previously, the Schwalbe lab was able to conduct these experiments for *E. coli* polymerase but not with the more demanding *B. subtilis* polymerase. From this work, we show different transcription rates and utilization of different pause sites. This has not been done before.

*3) The authors use separate oligonucleotides that act as surrogates for the actual base-paired interactions that occur in a unimolecular riboswitch. It is not clear that these interactions will have kinetic properties that accurately reflect analogous interactions that occur in the natural construct during RNA synthesis. If not, then the author's conclusions (such as those made in the eighth paragraph of the Results) might be flawed.*

*4) Related somewhat to 3: The associations between PA and T and between T and H are bimolecular reactions in the NMR experiments. However, during co-transcriptional folding, these associations are intramolecular reactions. It is not clear in the manuscript whether the authors have considered differences between these two types of reactions and corrected the NMR-determined associations rates for simulation. Further, the authors did not provide clear descriptions in this manuscript how the simulation was performed, how the pausing was implemented, and what rates were used. For example, t0,1~5s and t0,2~61s were labeled for ligand binding at the aptamer in Figure 5a. These values seem to be calculated from NMR determined k1 and k2 values. Again since the association rate is ligand concentration dependent was it corrected for simulation?*

Clearly, our experimental setup does not completely reflect the co-transcriptional RNA folding scenario in vivo. Instead, we present the structural basis for folding of the different transcription intermediates, which occur during riboswitch transcription in the cell. We show that the individual base-pair interactions of hybridized oligos represent the RNA structural motifs of the unimolecular RNA of the corresponding size. At the RNA concentrations used for NMR (>0.1 mM), we are confident that we mimick a scenario very close to the unimolecular situation. In a previous study (Warhaut et al., NAR 2017), we could show that for translational riboswitches, the aptamer domain and the expression platform are conformationally decoupled. Therefore, the intramolecular effect in RNA refolding is small. Further: our data from biophysical studies, mainly NMR, are fully consistent with the single-round transcription experiments that we can conduct under full control. This consistency makes us confident that our data will advance the field.

The values in the simulation that have to be adapted for concentration are accordingly adapted to match cellular concentrations comparable to Buckstein 2008, as referenced in the text.

*5) One of the major concerns of the current study is how transcription intermediates and pause sites were identified. It is possible that a transcription intermediate gives an aborted product in multi-round transcription, but the appearance of an aborted product in multi-round transcription can be a result of many other factors. The appropriate approach for identifying a transcription intermediate is to measure accumulation in time-resolved single-round transcription assays, such as what the authors have done to confirm pause site 2 (PS2). In this regard, in contrast to PS2, the identity of pause site 1 (PS1) is not clear, as no transcription intermediate at PS1 was detected in the time-resolved single-round transcription assay. However, in this manuscript, PS1 has been a critical intermediate for designing truncated riboswitch constructs, performing kinetic measurements, and simulating co-transcriptional pathways (see below). If PS1 is a true pause site, the authors need to clearly confirm the presence of PS1 and characterize its pausing properties.*

In order to identify the transcription intermediates, we performed time-resolved single-round transcriptions with the *E. coli* and the *B. subtilis* RNAPs in collaboration with the Landick lab. These assays gave distinct signals for the run-off transcript (or full length), for premature transcription termination at the terminator (GswPATH), and the pause-sites PS2 and PS1. In addition, we could identify further pause-sites (RNA77 and RNA95 for *E. coli* and RNA77 and RNA90 for *B. subtilis*). For both RNAPs, the ratio between full-length and terminator varied in a ligand dependent manner and the intensities of PS1 were higher than for PS2. We therefore conclude that PS1 plays a more crucial role for the co-transcriptional pathways. However, due to its strong pausing effect on the EC, which leads to a strongly halted complex, the determination of the pausing properties was rather difficult.

*6) Similar to the above concern, it is not appropriate to compare aborted products in multi-round transcription reactions to evaluate transcription intermediates between E. coli transcription and B. subtilis transcription. For example, there are five major products in the E. coli transcription, but there are least eight major products shown in the B. subtilis transcription. If aborted products were all transcription intermediates, this would suggest there are many more pause sites for the B. subtilis polymerase than for the E. coli polymerase, which is not likely. To properly evaluate transcription systems, the authors need to perform time-resolved single-round transcription assays and compare differences in transcription intermediates.*

We share the concerns of the reviewers and therefore performed time-resolved single-round transcription assays, as mentioned above, which revealed the nature of the transcription intermediates. We could show that PS1 plays an important role in the transcriptions using both, the *E. coli* and the *B. subtilis* RNAPs.

*7) In the figure for simulation, the authors labeled pausing at PS1 but did not label pausing at PS2. In the manuscript, the authors specifically discussed pausing at PS1 but did not discuss pausing at PS2. The experimental data identified PS2 but did not identify PS1. It is not correct to simply assume the presence of PS1 and ignore PS2. In addition, it is also not clear whether the half time at PS2 was used in the simulation, or whether it was used for PS1.*

We share the concerns of the reviewers and therefore performed time-resolved single-round transcription assays, as mentioned above, which revealed the nature of the transcription intermediates. We could show that PS1 plays an important role in the transcriptions using both, the *E. coli* and the *B. subtilis* RNAPs.

*8) Similar to the above concern, since PS1 was not identified in the* in vitro *assay, while mutating PS1 sequences do affect* in vivo *gene expression, it is difficult to evaluate these influences as mutation-induced changes in pausing or other factors, such as folding. The authors need to clearly confirm the presence of PS1 and may also need to characterize pausing properties of these mutations.*

We could identify PS1 in the newly performed in vitro assays and determine its role for the regulation mechanism.

*9) Regarding the Bacillus and E. coli RNAPs: These enzymes are quite different, and they are not considered to be indistinguishable on the basis of their pause kinetics. However, if the authors have new information on their comparative properties, they need to reference it. The authors are using E. coli RNAP on a B subtilis riboswitch, which is quite ok, but they need to be clear that they are measuring properties of EC RNAP.*

As outlined above we have performed optimized transcription assays (static and time resolved) using *E. coli* and *B. subtilis* RNA-polymerase, only the PS found for BS and their properties were included in the simulation of the riboswitch folding trajectory.

[Editors' note: further revisions were requested prior to acceptance, as described below.]

*[…] 1) The authors wrote in their response that there are no quantitative models to describe transcriptional regulation by riboswitches. This statement is not correct. While the kinetic model established in the current study represents one of the few quantitative models and has its own merits in advancing the understanding of transcriptional regulation by riboswitches, a similar regulatory model with quantitative kinetic parameters has been previously reported for the FMN riboswitch (Wickiser et al., 2005). The authors need to revise their Introduction to be consistent with the literature.*

We thank the reviewers for this comment. In the previous work, the effect of transcription speed and pausing on the FMN riboswitch from *B. subtilis* was analyzed and it was postulated that transcription speed plays a major role in the regulation mechanism of certain riboswitches. However, the K_D_ values were measured by comparing the termination rates under different conditions. Therefore, only the overall apparent rates were determined. In addition, no transcription rates were quantified. In our work, we combine measuring exchange rates and K_D_ values at nucleotide level with quantified transcription and pausing rates. We can therefore determine the conformational with base pair resolution and gain a more detailed view. In addition, we use a simpler transcription system consisting of only the template, the RNAP and the RNA. This overcomes the problem that additional transcription factors could alter the interaction between RNAP and the riboswitch which was also mentioned in Wickiser et al., 2005.

*2) One major concern of the original manuscript was the application of multi-round transcription assays to evaluate transcription intermediates. In the revised manuscript, the authors have performed time-resolved single-round transcription assays to properly establish transcription intermediates. However, despite having these appropriate measurements, it is somewhat surprising that the authors have kept applying the multi-round transcription data to evaluate transcription intermediates (Figure 1E). The authors need to update the text and figure with proper data from their new single-round transcription assays.*

We thank the reviewers for this comment. When we synthesized the full-length riboswitch sample by T7 mediated in vitro transcription, the analytic PAGE showed several abortion products. NMR data of the full-length riboswitch revealed that it was unable to switch. When we investigated this phenomenon, we realized that the abortion fragments on the gel could correspond to stable transcription intermediates, which caused the T7 RNAP to abort transcription. Comparison to the *E. coli* RNAP showed similar results. These results were taken into account when the shortened riboswitch and the trans added RNAs were designed for the time resolved NMR experiments.

*3) In the revised manuscript (Results, fifth paragraph), the authors changed the length of one of the transcription intermediates from the original 125 nucleotides to 140 nucleotides. Since this RNA fragment was proposed to represent pause site 2 (PS2), this new length will mark PS2 around nucleotide A150, which is different from PS2 at nucleotide U141 shown in both Figure 1A and Figure 4. The authors need to make sure the location of PS2 is consistent throughout the manuscript.*

We thank the reviewers for this comment. Compared to the wild type sequence, the NMR construct is missing 10 nt and therefore starts with G10.

*4) Similar to the above concern, the labels for PS1 and PS2 are confusing in the new Figure 4. For example, PS1 is labeled as 110; however, the RNA band marked as PS1 clearly migrates between the 110 and 123 bands of the sequencing ladder. In addition, the RNA band marked as 141:PS2 migrates right below the 147 band of the sequencing ladder in the Bs RNAP assay. However, in the Ec RNAP assay, there are no apparent RNA bands at similar locations; instead, there is a major RNA band that seems to migrate between the 123 and 147 bands of the sequencing ladder. The authors need to have proper labels for the transcription intermediates in both the Ec RNAP assay and the Bs RNAP assay.*

One transcription intermediate was identified as 110 which migrates between the bands 110 and 123 of the sequencing ladder. In order to clarify the length of this fragment, a 3’-mapping of the transcriptions was performed using 3’-deoxynucleotides (Figure 4—figure supplement 1). The gel shows several transcription abortion products which end with a 3’-deoxy U and which migrate slightly faster than the 110 RNA fragment. When compared to the sequence, this poly U sequence corresponds to the bases T107 to T112. However, it can’t be clearly stated on which nucleotide the 110 RNA ends. We’ve therefore chosen to call this fragment 110. For the simulations and for the transcription rate determination, the results don’t change when the length of this fragment is varied from 107 to 112 nucleotides. We apologize for the badly indicated band at 141 nt. The label in the figure was displaced. We changed it to the original position.

*5) In the new supplementary Table 8 (Supplementary file 1), the authors reported transcription rates and pause site characteristics from time-resolved transcription assays. While the majority of the reported values are reasonable, some measurements are quite confusing. For example, the average appearance time of PS2 (141 nts) is shown to be 3.0 seconds in the third table. However, this time value is even less than the average appearance time of the shorter RNA77 (77 nts) fragment (13.2 seconds). In addition, an appearance time of 3.0 seconds is also less than the shortest time point (5 seconds) of the time-resolved assay. Another outlier is the average appearance time of Gsw-PATH, which is shown to be 775.9 seconds in the fourth table. However, it can be seen in Figure 4 that the RNA band for Gsw-PATH starts appearing around 20 – 30 seconds for the Bs RNAP assay. In addition, this appearance time is more than the longest time point (600 seconds) of the time-resolved assay. The authors need to go through the tables and make sure these values are properly reported. If these outliers are authentic, the authors need to comment on them.*

We thank the reviewers for this comment and have improved discussion of the results in the paper. The mentioned values were determined from signal intensities close to the noise which is also indicated by the high error. However, the transcription speed for the *E. coli* RNAP was also determined in the previous version of this paper. The values were in accordance with literature and could therefore be considered for the simulations.

*6) One other concern of the original manuscript was the lack of details for the co-transcriptional folding simulation. While the authors have provided more details in their revised manuscript, a key aspect of the simulation remains not clear. The authors need to describe in the method how pausing was implemented in the simulation.*

We have corrected this and included the details of simulation in the Materials and methods section by including the following paragraph:

“Two sets of simulations were performed, in the first set the transcription rate was adjusted to the experimental value for transcription without any pausing events, while the second set simulated a pausing event between states PA/^lig^PA and PAT/^lig^PAT. […] Thereby, the reduction of the transcription rate over the pause site corresponds to a factor of ≈70 in the simulations.”
